# Partisan aesthetics: political orientation modulates aesthetic judgments of propaganda posters

**DOI:** 10.3389/fpsyg.2026.1803013

**Published:** 2026-08-12

**Authors:** Erkin Özmen, Gregor Uwe Hayn-Leichsenring

**Affiliations:** Institute for Anatomy I, University Hospital Jena, Jena, Germany

**Keywords:** experimental aesthetics, cognition, gist perception, quantitative image properties (QIPs), political orientation, propaganda posters

## Abstract

This study explores how aesthetic perceptions of WWII propaganda posters are modulated by cognitive processing of political information and viewers’ Political Orientation, drawing on Redies’s unified model of aesthetic experience. In an experiment involving 135 participants, each subject rated a series of randomly drawn Allied and Axis propaganda posters under two exposure conditions: Restricted Exposure (RE) and Unrestricted Exposure (UE). We analyzed ratings in each exposure condition in addition to the between-exposure change in aesthetic appeal, focusing on effects of stimulus-level political context and participant-level political orientation (Left, Center, Right) and gender. With Linear Mixed Modeling, we analyzed key factors within the wider context of stimulus- and participant-level variables. Our findings show that Axis posters lose appeal with extended exposure, suggesting that prolonged engagement tends to lower subjective aesthetic appeal—specifically for these stimuli. Political Orientation and Sex further influenced perception: Left-wing and centrist participants exhibited significant rating shifts between RE and UE conditions, while right-wing individuals did not. Notably, we observed a marked difference in how appeal changed between males and females. Overall, our study provides evidence that political orientation, gender and semantic image content influence subjective ratings of aesthetic appeal.

## 1 Introduction

### 1.1 Art and propaganda

“*All art is propaganda*.”**—**(Sinclair, 1925).

The emergence of mass media in the early 20th century coincided with great social upheaval and geopolitical tensions culminating in two World Wars. The impact of sustained industrialized conflict on civilian and military life drove governments to engage in concerted propaganda efforts to maintain support for the war (Artinger, 2000; Chambers, 2012; Fondren, 2021). Governments simultaneously recognized the potential of propaganda images to convey their beliefs (Eken and Talug, 2023; Fondren, 2021). In the absence of modern mass media, propagation of images relied on print (Artinger, 2000; Connelly, 2019; Eken and Talug, 2023; Lewis, 2004; Weitz, 2000). This, in turn, generated demand for dedicated propaganda art.

According to O’Donnell and Jowett, the term propaganda, in its most neutral sense, refers to the dissemination or promotion of ideas (O’Donnell and Jowett, 1992, p. 2). Orwell, by contrast, ascribes a far more ambitious aim to propaganda in his fictional novel 1984: Here, Oceania’s Ministry of Truth controls all information and rewrites history according to the party narrative—thus altering the very reality of its citizens. In line with this, Staal defines the purpose of propaganda art as shaping the visual reality according to the interests of power structures (Staal, 2018, p. 10).

Consequently, great importance was attributed to the aesthetic form of propaganda posters, as evidenced by the considerable efforts made to craft visually appealing images: large staffs of prominent artists and graphic designers were employed throughout WWII in state-sponsored propaganda design bureaus, with prominent examples including the Propaganda atelier under the auspices of the German Ministry of Propaganda (Weiss, 2021) and the United States Office of War Information (OWI) with its in-house Bureau of Graphics (Witkowski, 2003). These studios supplied high-quality paintings and drawings to their countries’ war efforts, leveraging aesthetics for persuasion.

Despite considerable debate on the utility, morality and inherent dangers of propaganda in the aftermath of WWI (Creel, 1920; Lasswell, 1927; O’Donnell and Jowett, 1992), the interwar period was marked by a resurgence of increasingly sophisticated state-sponsored propaganda efforts in the lead-up to the Second World War. Soon after outbreak, public spaces came to be dominated by war-posters aimed at mobilizing the masses (Chambers, 2012; Connelly, 2019; Eken and Talug, 2023; Fondren, 2021; Weitz, 2000), with large-scale poster campaigns on both sides of the conflict featuring common themes such as enlistment, counter-espionage, productivity and public morale (Gautam, 2012; Weiss, 2021; Witkowski, 2003).

While the design and messaging strategies of war-posters have been studied extensively by art historians and political scientists alike (for an overview, see Connelly, 2019; Witkowski, 2003), the mechanisms underlying their subjective aesthetic appeal—especially how they resonate with audiences depending on their political views—remain largely unexplored.

### 1.2 Neural correlates of aesthetic experience

One major challenge in experimental aesthetics is defining whether stimuli possess an intrinsic “significant form” that elicits beauty or if aesthetic experience is primarily context-dependent. Formalist theories propose that aesthetic experience relies on formal features that are universal to beautiful visual stimuli. Conversely, contextual theories suggest that aesthetic experience depends on the processing of explicit information, i.e., image content and context (Redies, 2015). Further insight is offered by recent advances in functional neuroimaging that have empowered research on the neural underpinnings of aesthetic experience as well as political and social psychology. Interestingly, aesthetic judgments trigger activation in brain regions that have likewise been implicated in moral and political decision-making. Converging evidence indicates that this shared neural network prominently includes the medial prefrontal cortex (mPFC), a central hub for evaluative judgments and subjective valuation (Jacobsen et al., 2006; Jost et al., 2014; Sevinc and Spreng, 2014); temporal regions (temporal pole and temporoparietal junction), associated with complex visual, semantic and socio-emotional processing (Garrigan et al., 2016; Jacobsen et al., 2006; Sevinc and Spreng, 2014); the cingulate cortex, which is involved in monitoring cognitive conflict and determining the personal relevance of stimuli (Garrigan et al., 2016; Jost et al., 2014; Vartanian and Skov, 2014); and the amygdala, with its well-established role in basic affective and attentional processes integrating emotionally salient information across evaluative domains (Di Dio and Gallese, 2009; Jost et al., 2014; Sevinc and Spreng, 2014). These shared neural correlates are also consistent with real-world phenomena such as the ‘Beauty-is-Good’ stereotype, wherein aesthetic perception interacts with socio-moral cognition to assign positive character traits to attractive individuals (Ferrari et al., 2017; Griffin and Langlois, 2006).

### 1.3 Cognitive and perceptual mechanisms in aesthetic experience

In line with these insights, an examination of the aesthetic appeal of propaganda posters must account for contextual processing in addition to formal perceptual features. To this end, we utilize the unified model of visual aesthetic experience (UMAE) proposed by Redies (2015). Based on the triad of cognition, perception and emotions (Chatterjee and Vartanian, 2014), the UMAE reconciles formalist and contextual theories by distinguishing between two independent, parallel channels: Perceptual processing and cognitive processing.

The perceptual channel operates through a fast bottom-up mechanism that processes the form of visual stimuli. This channel relies on features such as symmetry, complexity, and self-similarity, which previous studies have shown may be influenced by evolutionary the predisposition of the human visual system, reflected in consistent aesthetic responses to certain Quantitative Image Properties (QIPs) (Hayn-Leichsenring et al., 2017; Redies, 2015; Schwabe et al., 2018; Redies et al., 2025).

By contrast, the cognitive channel operates as a top-down mechanism, integrating semantic content and context with the prior knowledge and expectations of the beholder through a personal cultural filter. This filter is a mental construct shaped by individual experience within a certain cultural context; when its settings are compatible with an artwork’s contextual information, it permits ‘cognitive mastering’ (Redies, 2015). In the current study, we posit that a viewer’s political orientation and ideological alignment function as critical components of this personal cultural filter, shaping their engagement with WWII propaganda posters.

Since the channels described in the model appear to be processed differently in the brain, we assume that they can also be differentiated behaviorally. Previous studies showed that gist experiments can be used to isolate the effect of QIPs from semantic content (Mullin et al., 2017). In detail: The effect of low-level image properties (i.e., complexity, anisotropy, self-similarity, and color) can be extracted very rapidly. Therefore, ratings for low-level aesthetic appeal obtained under Restricted Exposure should be relatively similar to ratings obtained under Unrestricted Exposure condition ratings. Conversely, extraction and processing of semantic image content and text is slower and can, therefore, only influence ratings of aesthetic appeal in longer exposure conditions. Based on these findings, we speculate that the perceptual processing channel is more prominent in Restricted Exposure, whereas the cognitive processing channel is more prominent in Unrestricted Exposure.

### 1.4 Research aims and hypotheses

Building on the theoretical foundation of the UMAE, our study investigates how the aesthetic perception of propaganda art changes once observers become aware of its semantic (= political) content. Our hypothesis includes the assumption that political attitudes of participants influence aesthetic perception. Based the model of aesthetic experience, we hypothesize that the perceptual processing of visual features in propaganda posters partly predicts aesthetic evaluation of these stimuli across participants. Furthermore, we speculate that image semantics and contextual cues in propaganda posters will influence cognitive processing and, subsequently, subjective aesthetic appeal of these stimuli, depending on the viewer’s political views, resulting in divergent aesthetic evaluations.

To analyze the effect of perceptual and cognitive processing on aesthetic judgements in a political context, we decided to compare two different ratings within each subject: The initial, time-restricted exposure rating isolated the perception of stimulus-form from context (gist perception), thus representing aesthetic appeal under predominant activation of the perceptive processing channel. A second rating obtained after Unrestricted Exposure to the same stimulus, would reflect potential influence of cognitive processing. We predict that aesthetic ratings of propaganda posters in Restricted Exposure are not affected by political views because semantic content is not perceivable in such a short time. Conversely, we hypothesize that ratings in the Unrestricted Exposure condition will diverge depending on the observers’ political views. Overall, our study seeks to further the understanding of how visual-perceptive and political-cognitive factors interact, offering insights into the mode of functioning in human aesthetic perception.

## 2 Methods

### 2.1 Materials and methods

#### 2.1.1 The propaganda database

In our study, we used digitized images of wartime posters as stimuli. We limited the scope to German and Western Allied posters from World War II. Focusing on this specific wartime context limited variation in artistic styles and thematic content, thus facilitating better comparability between posters.

We collected 635 digitized war posters from several publicly accessible online archives: Scans of 255 German-issued posters were obtained from the German Federal Archives’ (Bundesarchiv, 2025) digital picture database and the Netherlands institute for War Documentation. 380 Western Allied posters, predominantly British- or American-issued, were collected from the Princeton University Poster Collection at the Smithsonian Institution Archives.

**Figure 1:**
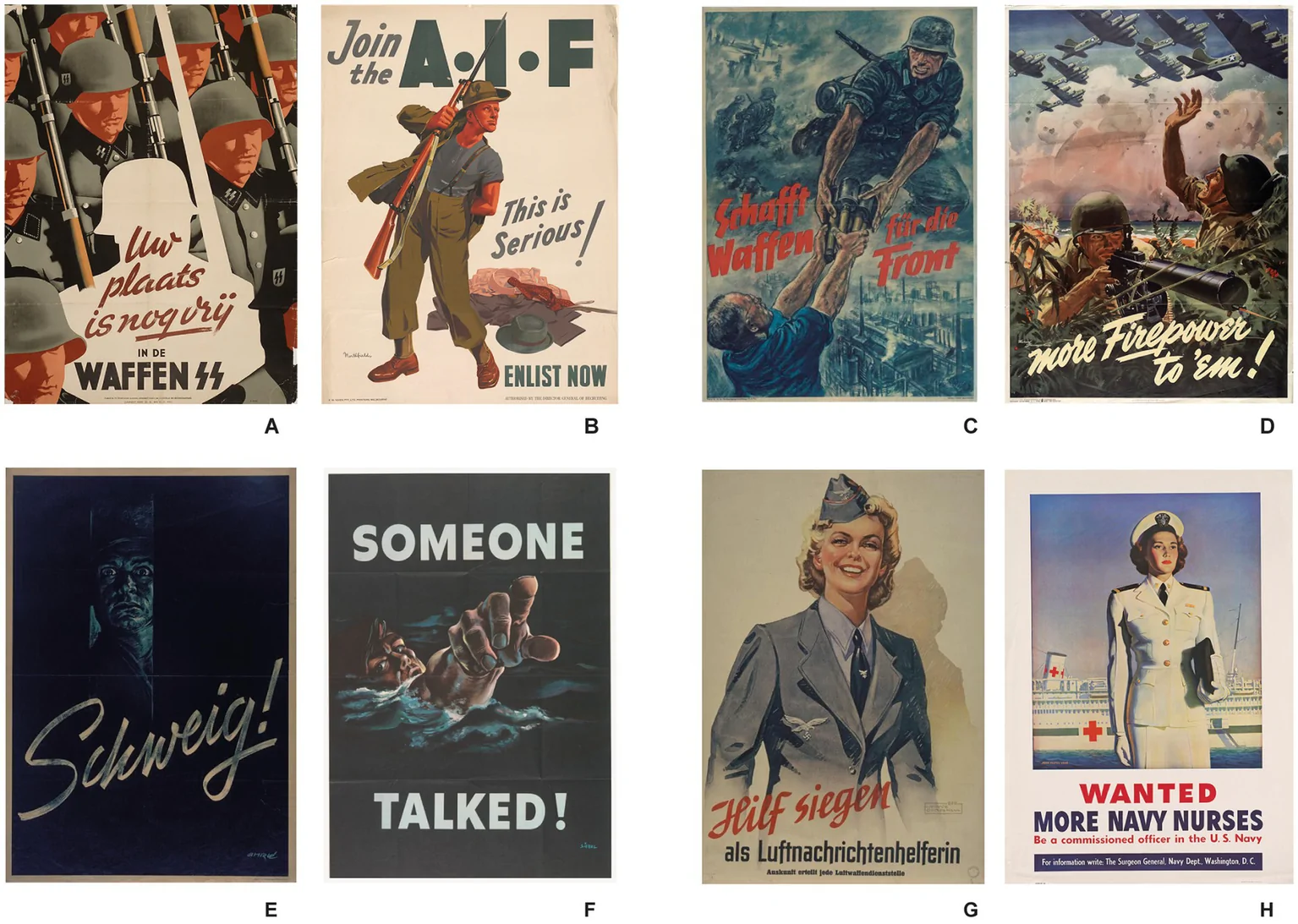
Example Posters from the Propaganda Data Base. **(A,B)** show enlistment posters. **(C,D)** address industrial productivity. **(E,F)** show typical examples of “loose-lips”-propaganda. **(G,H)** show enlistment posters addressed specifically at women. Images sources: NIOD Institute for War, Holocaust, and Genocide Studies (2025): AG/00087 (Affiche, Lithografie), Oorlogsaffiches NIOD 1933–1946, NIOD/KB **(A)**; Ordnance Department, U.S. Army, ca. 1939–1945. Courtesy of the State Archives of North Carolina, WWII Posters Collection **(D)**; Siebel, Frederick, 1942; University of North Texas Libraries, UNT Digital Library **(F)**; Princeton University Poster Collection, Archives Center, National Museum of American History (2025) **(B,H)**; German Federal Archives (Bundesarchiv, 2025): BArch Plak 003-023-079, Weber, Fritz R. **(C)**, BArch, Plak 003-027-024/Ahrlé, René **(E)**, BArch Plak 003-025-017, Biedermann, Walter **(G)**.

**Figure 2:**
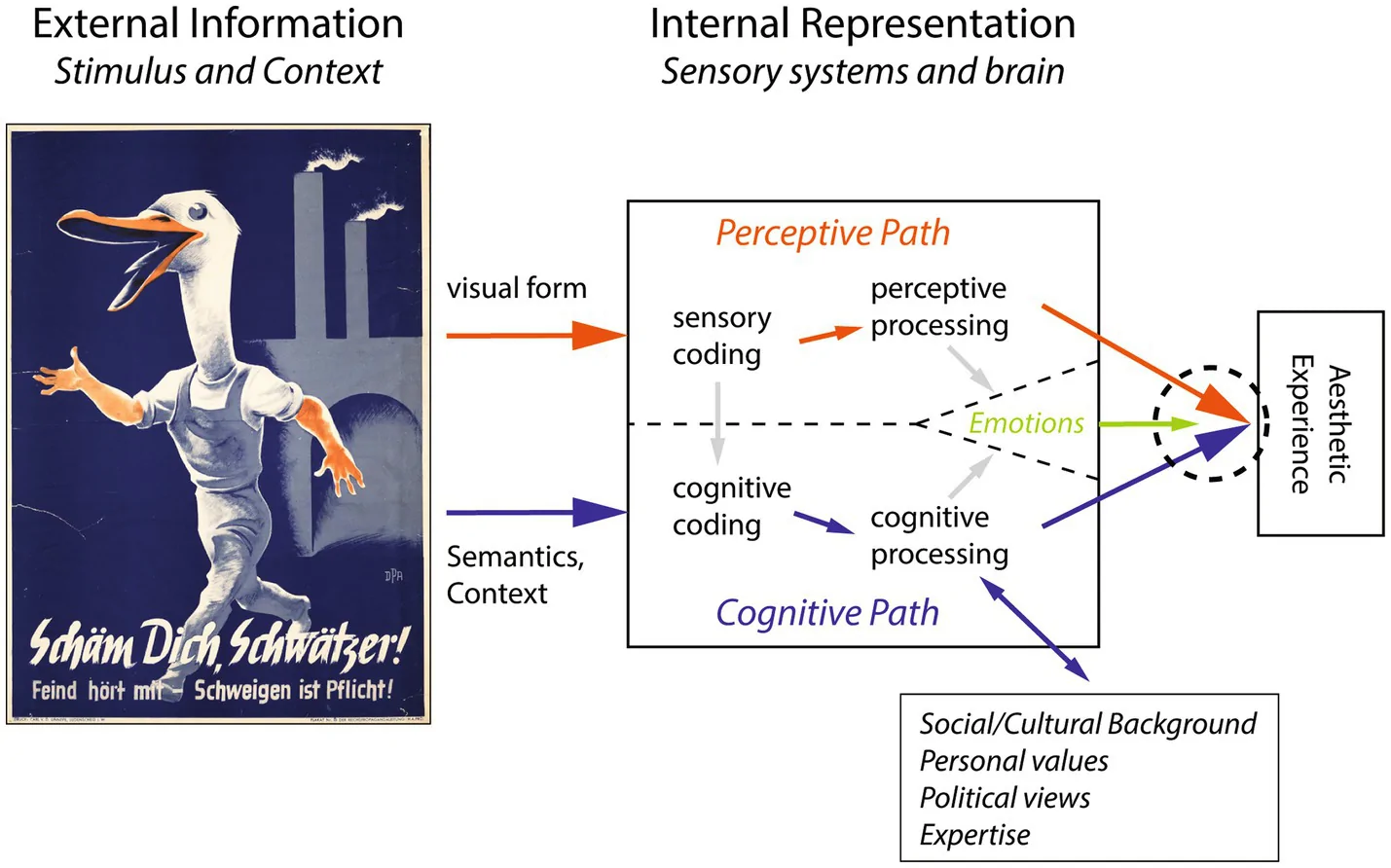
Model of aesthetic experience as proposed by Redies (2015) adapted for wartime propaganda posters. Image source (poster): AG/00432 (Affiche, Lithografie), Oorlogsaffiches NIOD 1933–1946, NIOD/KB.

Images were then categorized by hand (E.Ö.) according to image type (photograph, drawing, collage), origin (i.e., the publishing faction) in addition to a detailed set of classifiers (see Table 1). We calculated four Quantitative Image Properties (QIPs) that have been associated with aesthetic images in previous research to control for their potential influence on ratings (Redies et al., 2025): HOG (histogram of gradients) complexity, HOG anisotropy, PHOG (pyramidal histogram of oriented gradients) self-similarity and color values (HSV, hue, saturation, value) (see Figures 1, 2).

**Table 1:** Counts of stimulus classifiers in stimulus pool and experimental sample, by stimulus category (publisher).

Classifier	Level	Stimulus pool^a^			Sampled^b^		
		*n* Allied	*n* Axis	*n* Total	*n* Allied	*n* Axis	*n* Total
Tone	Positive	32	27	59	1,017	959	1976
	Negative	8	13	21	333	391	724
Intent	Recruitment	14	6	20	413	139	552
	Productivity	7	9	16	283	200	483
	Warning	8	10	18	330	318	648
	Morale	11	15	26	324	693	1,017
Theme	Military	19	14	33	586	539	1,125
	Workforce	12	9	21	440	184	624
	Society	9	17	26	324	627	951
Featured	Symbols	7	3	10	175	138	313
	Combat	9	7	16	265	299	564
	Gadgets	2	2	4	99	70	169
	People	22	28	50	811	843	1,654

a *n* = 80 stimuli.

b *n* = 2,700 observations.

##### 2.1.1.1 HOG complexity

Complexity refers to the amount of detail or variation in an image. A complex image has many different features or structures, whereas a simple image has relatively few. Here, we used a MATLAB algorithm to measure objective complexity based on gradient strength.

##### 2.1.1.2 HOG anisotropy

Anisotropy refers to the degree to which an image exhibits directional dependence. Anisotropic images exhibit luminance gradients that point in the same direction, while in isotropic images, gradient orientations are equally distributed (Redies et al., 2012).

##### 2.1.1.3 PHOG self-similarity

Self-similarity refers to the extent to which an image contains similar patterns at different scales or levels of detail. Self-similar images have structures that repeat themselves at different levels of magnification (Amirshahi et al., 2012; Mullin et al., 2017).

##### 2.1.1.4 HSV color space

The HSV (Hue, Saturation, Value) color model is structured as a cylindrical representation that separates chromatic information from brightness. In this model, *Hue (H)* represents the type of color, corresponding to the wavelength on the visible spectrum, measured as an angle between 0° and 360° on a color wheel. *Saturation (S)* defines the color’s purity or intensity, ranging from 0 to 1 (fully saturated). *Value (V)* indicates the brightness of the color, ranging from 0 (black) to 1 (maximum brightness) (Al-Hetar et al., 2019).

The whole dataset will be made publicly available on OSF for future research. The authors confirm that the data supporting the findings of this study are available within the article and/or its Supplementary material.

For the online study, we selected 80 images from the dataset. Using the previously described categorization scheme, we chose posters featuring similar topics and themes from both factions to obtain a representative pool of stimuli for our experiment. QIP values for posters included in the study were within one SD of the dataset average. Image height was standardized at 750 pixels with a resolution of 96 dpi.

### 2.2 Study design

Initially, 157 volunteers of various educational and cultural backgrounds were recruited via Prolific^1^ to participate in an experiment conducted on the SosciSurvey online platform.^2^ Of those, five subjects exited the experiment before completion and were excluded. Another seven datasets were excluded for scoring beyond recommended cutoffs on Sosci Survey’s quality metrics (completion speed and relative speed index) and 10 more subjects were excluded either due to low variability in ratings, or failure of the embedded attention check, as these were taken as indicators of poor task-engagement. Ultimately, we analyzed data collected from 135 participants in our study (see Table 2).

**Table 2:** Recruited participant and their political alignment.

Sex	Left	Center	Right
Male (*n =* 86)	35	13	38
Female (*n =* 49)	29	7	13
Full sample (*n =* 135)	64	20	51

**Figure 3:**
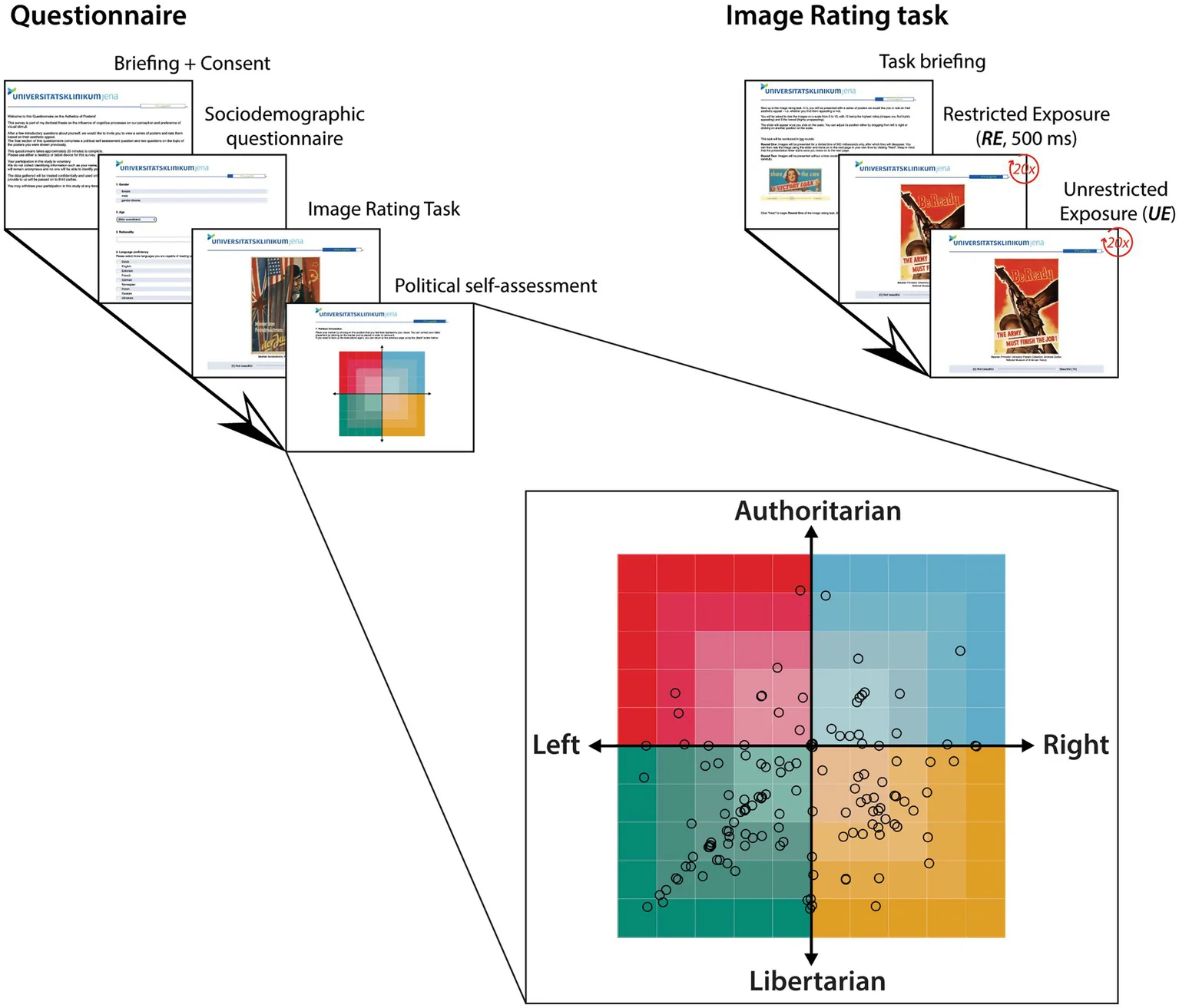
Design of the online questionnaire.

For a graphic representation of the questionnaire, see Figure 3. The study was conducted in accordance with the principles of the World Medical Association Declaration of Helsinki and was approved by the Ethics Committee of Jena University Hospital (reference no. 2022-2542). Upon recruitment, participants were presented with a dedicated briefing page placed at the beginning of the questionnaire. This briefing page provided participants with a detailed explanation of the study aims and information on their right to withdraw at any time without consequences, followed by an informed consent form. Participants who declined consent automatically exited from the experiment, while those who provided consent proceeded to the sociodemographic section of the questionnaire. Next, participants were briefed on the upcoming image rating task (IRT) on a dedicated briefing page explaining the exposure conditions and interface. Participants were instructed to rate images on a sliding scale from 0 (not beautiful) to 100 (beautiful). Clicking on the scale revealed a slider, with the corresponding numerical rating displayed directly above; further adjustments to the rating could be made by dragging the slider along the scale. Participants advanced to the next stimulus by clicking a “Continue” button at the bottom of the screen. Each participant was presented with an individualized stimulus set of 20 posters, consisting of 10 posters drawn at random from the Allied pool and 10 from the Axis pool for balanced exposure to both stimulus categories (i.e., *Publishers*), thus ensuring comparability across participants while accounting for the full diversity of the stimulus pool. Limiting each participant to a subset of 20 out of the total 80 available stimuli helped keep the task brief, thereby reducing the risk of disengagement to maintain data quality in an online, limited-control environment.

The IRT employed a repeated-measures design, where each participant rated their full stimulus set twice: first under a *Restricted Exposure* condition, (*RE*, rapid presentation time of 500 ms), followed by an *Unrestricted Exposure* condition (*UE*, unlimited viewing time). These two blocks were presented consecutively, with all 20 images appearing in a newly randomized sequence during the second block. (see Figure 3).

The presentation time in the RE condition (“gist perception”) would ideally be around 100 ms, however, trial runs showed that, depending on device and internet connection, in an experimental setting of 100 ms, true exposure time could be severely restricted to the point of the stimulus not becoming visible at all. Through further trials, we determined that 500 ms provided an adequate compromise between short presentation time and technical feasibility.

An attention check mimicking the image rating task was embedded between stimulus presentations during the UE block of the experiment.

Upon completion of the IRT, participants were directed to the political self-assessment (PSA). This section was positioned at the end of the questionnaire to avoid influencing aesthetic ratings through priming effects. For the PSA, we used an adapted version of the Political Compass: a two-dimensional model of Political Orientation with a Left–Right *X*-axis and an Authoritarian–Libertarian *y*-axis.

Preliminary data exploration revealed a highly skewed distribution of responses along the y-axis, with clustering in the centrist and libertarian regions and few respondents in the authoritarian region. This pattern may reflect a general tendency among participants in our sample to project Political Orientation primarily onto the more familiar Left–Right axis. Moreover, we found no meaningful correlation between y-axis values and any of the rating variables in preliminary analyses. Based on this, we limited all further analyses to the Left–Right *X*-axis (see Figure 3).

**Figure 4:**
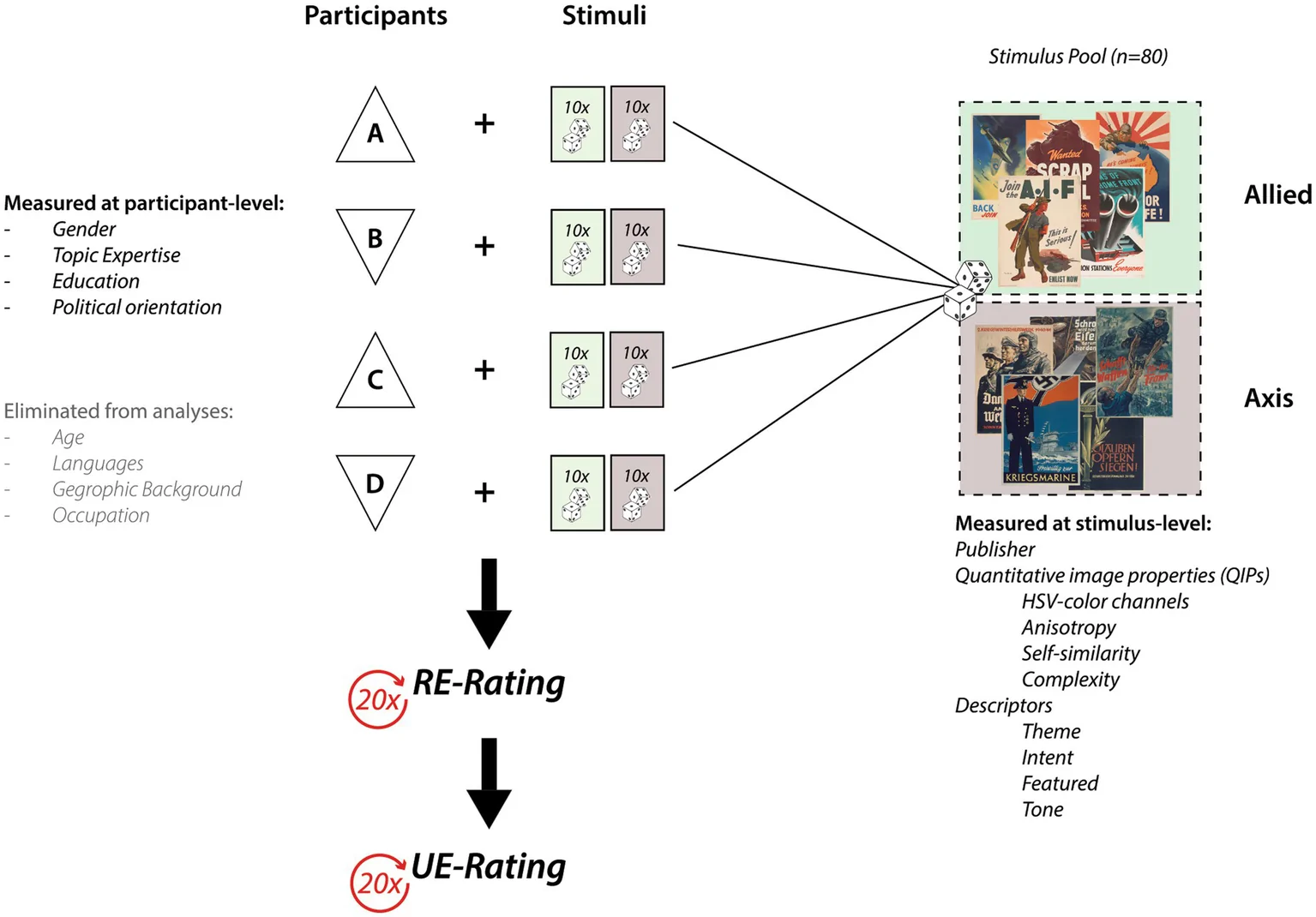
Stimulus distribution. 10 Stimuli were sampled at random from each category (Allied, Axis) of the Stimulus pool. Participants (A, B, C, D) then rated their full stimulus sets under both exposure conditions (RE and UE).

Before advancing to the PSA task, participants viewed a dedicated briefing page explaining the axes of the Political Compass. They were then asked to indicate their Political Orientation by placing a cursor on the chart. Responses were recorded on a continuous scale from −3 to +3 on each axis. For the *X*-axis (Left–Right), we classified participant with cursor placements below −0.25 as *Left-wing*, participant with values above +0.25 were assigned to the *right-wing* group and those with cursor placements that fell within an uncertainty region between −0.25 and +0.25—representing approximately 10% of the observed *X*-axis range—were classified as *centrist* (see Figure 4).

### 2.3 Statistical methods

To obtain a measure for the between-exposure effect, we computed *RatingDELTA* as the within-observation difference between Unrestricted Exposure (UE) rating and Restricted Exposure (RE) rating of a given participant-stimulus combination (i.e., *RatingDELTA = ReatingUE – RatingRE*). Positive values indicate that an increase in appeal going from RE to UE, whereas negative values indicate a loss of appeal.

For an initial high-level overview, we first aggregated data to participant means for our outcome variables *RatingRE*, *RatingUE* and *RatingDELTA*. For between-group comparisons, we compared means across groups via unpaired *t*-tests (two level factors: *Publisher, Sex*) or one-way ANOVA (three-level factor: *PolCat3*, i.e., Political group). For between-exposure comparisons, we performed one-sample *t*-tests, to determine whether mean *RatingDELTA* differed from zero (i.e., no between-exposure change) within groups defined by *Publisher, Sex*, *PolCat3* and combinations thereof.

**Figure 5:**
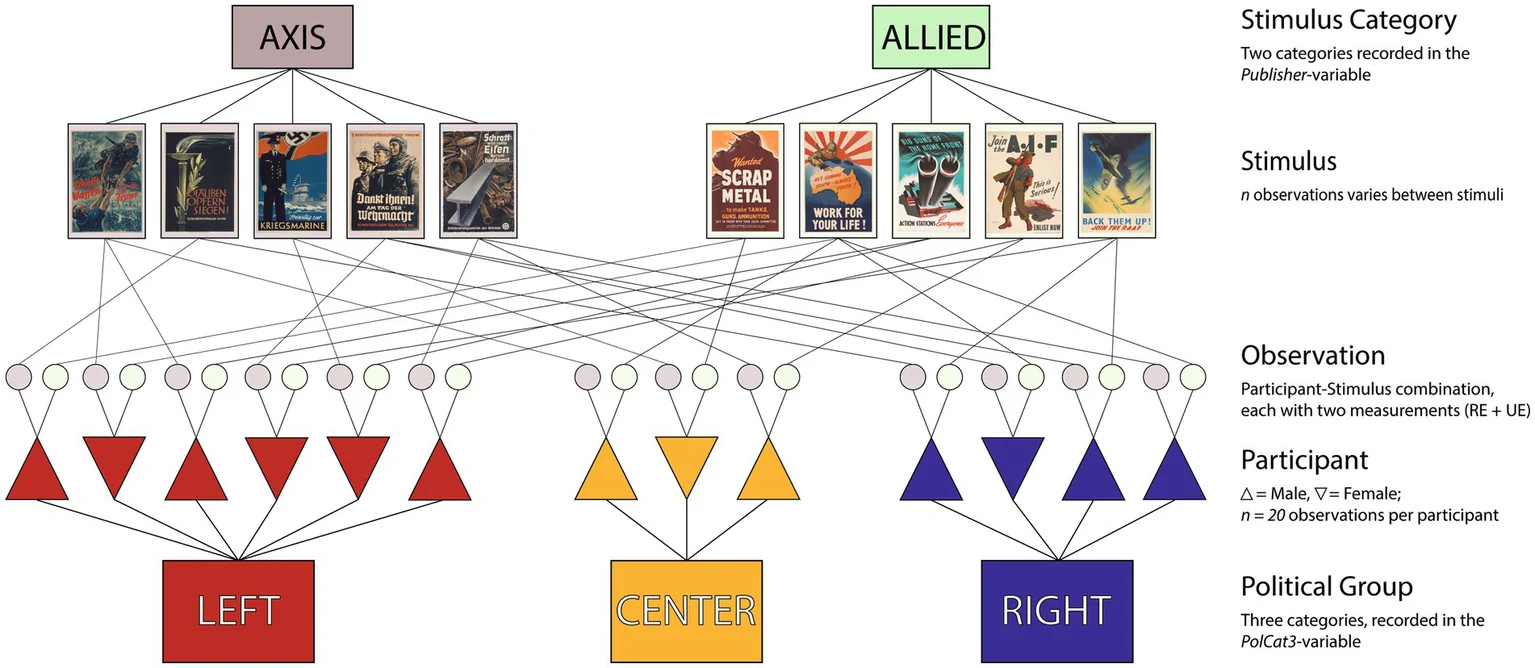
Visualization of data structure and hierarchy. Triangles represent male and (inverted) female participants; circles represent observations, of which two—one Allied and one Axis—are shown per participant. Our LMM applies a crossed random effects structure, where observations are clustered within Interview and Stimulus. To address our hypotheses, our model is aimed at obtaining coefficients for Political groups and stimulus categories; they are not “samples” from a wider range of possible, unobserved groups, they are the categories of interest. Hence, we do not nest the random effects of participant and stimulus in their respective higher-order cluster. Furthermore, since both, participants and stimuli, only ever belong to one group, the model assumes no residual group-level correlation beyond what is explained by the fixed coefficients for Political group and Stimulus category.

Second, we fitted linear mixed effects models (LMMs) to the original study data, thereby preserving information at the observation-level. Here, we directly model the between-exposure change (*RatingDELTA*) as a function of participant- and stimulus-level variables, thus providing a statistical framework that facilitates testing of our main hypothesis. Crucially, a LMM can be specified to accurately reflect the underlying data structure and hierarchy (see Figure 5) through its random effects. Further, simulation studies have demonstrated that LMMs fitted on unbalanced data sets are generally robust, provided that data are missing completely at random (MCAR) with respect to the parameters and response of interest (Schielzeth et al., 2020). As the missing data in our experimental sample depend neither on covariate values (i.e., missing at random, MAR) nor the response variable or unobserved predictors (i.e., missing not at random, MNAR), the MCAR assumption holds in our data set.

Following the recommendations in Yaremych et al. (2023) for hierarchical data structures, predictors were standardized prior to modeling. We distinguished three groups of predictors for model selection: (i) baseline control predictors, (ii) stimulus-level predictors, and (iii) subject-level predictors. Group (i) included *pmcGist*, a within-subject *z*-score centered at participant-means to control for the range-constraints, and Publisher. Given its central role in our experimental design, Publisher was retained in every candidate model as a baseline predictor of interest. Group (ii) comprised Quantitative Image Properties (QIPs) standardized at the grand mean, in addition to image descriptors, while group (iii) included key predictors such as Sex and Political Orientation (PolCat3), along with their interaction with Publisher. Predictors in groups (ii) and (iii) were allowed to compete freely during predictor selection to establish their significance within the model space; consequently, the resulting model structure represents an exploratory result of the study.

**Figure 6:**
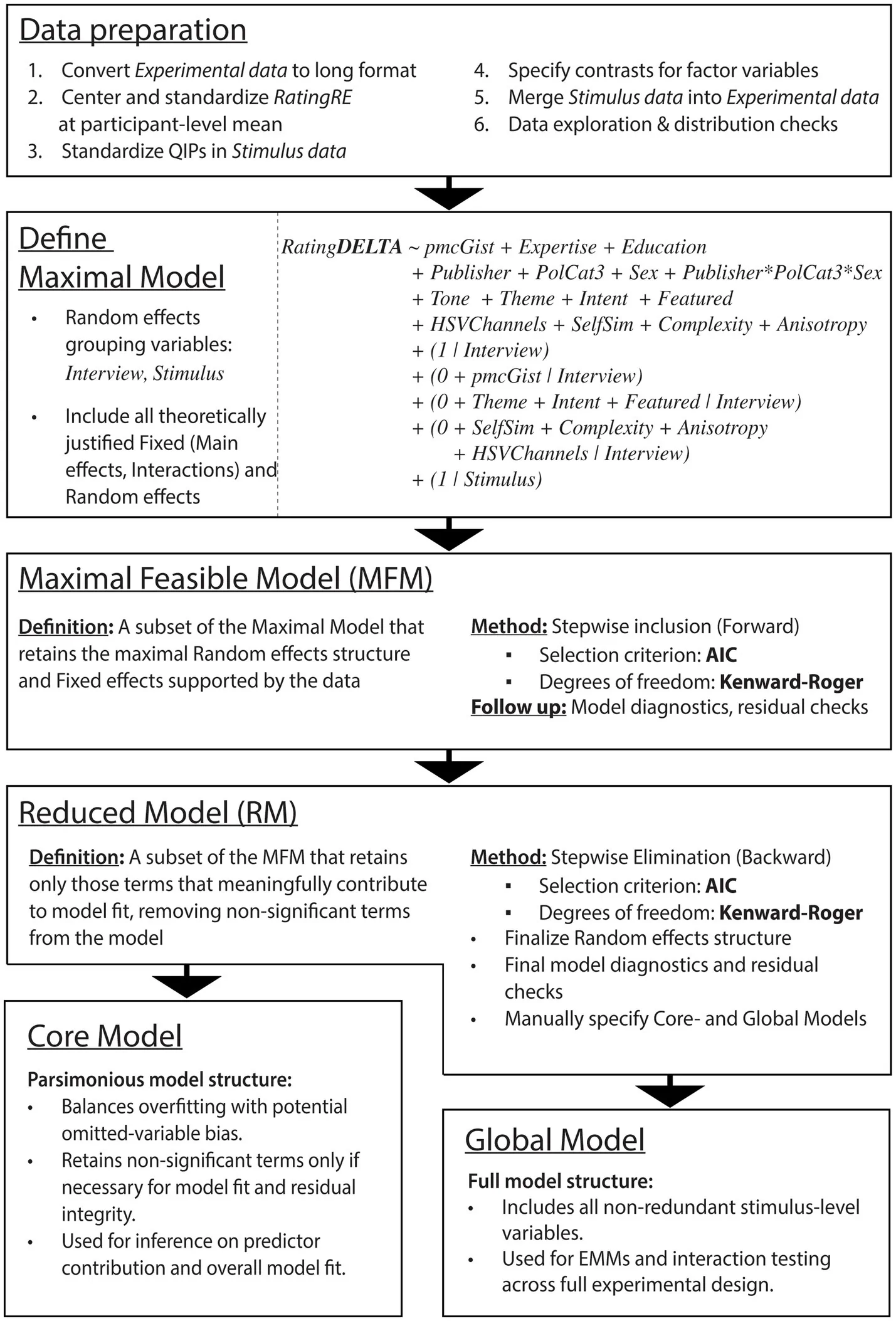
Flowchart of the model selection process.

Figure 6 provides an overview of our modeling pipeline. Initially, we specified Interview (participant) and Stimulus as random-effects grouping factors, reflecting the crossed repeated-measures design. Based on this, we defined a Maximal Model (MM) that included all theoretically justified fixed- and random effects terms to control for all potential confounds (Barr et al., 2013; Harrison et al., 2018). As the overparameterized MM did not converge, we derived a Maximal Feasible Model (MFM)—representing the most comprehensive subset of the MM that would still converge—from the MM (Bates et al., 2018, Matuschek et al., 2017), using iterative forward-selection facilitated through the *buildmer* function.

After performing model diagnostics and residual checks, we derived a Reduced Model (RM) from the MFM through backwards-elimination of non-significant fixed effects to obtain a parsimonious model that preserved model fit and explained variance, as recommended by Zuur et al. (2009). Model selection was guided by the Akaike Information Criterion (AIC), in line with recommendations by Harrison et al. (2018).

To arrive at the Core Model—the statistical framework for assessing the contribution of individual predictors—DHARMa-simulated residual diagnostics were employed to guide manual refinement of the RM. Non-significant terms—such as *V-Channel* or the Sex*PolCat3 interaction—were retained if their removal compromised model stability or introduced residual heteroskedasticity, which can signal missing predictors (Hartig, 2024). The resulting model structure represents an exploratory result of this study.

The Global Model was derived directly from the Core Model by reintroducing previously eliminated stimulus-level variables (e.g., *Theme*, *Intent*), thus ensuring that estimated marginal means (EMMs) were adjusted for the full complexity of the stimulus pool. A comprehensive explanation of the theoretical and technical aspects that informed each step of this process is provided in the Supplementary material. For diagnostic plots of all models discussed in this article, see Supplementary Figures 4–11.

The R program, version 4.4.1 (R Core Team, 2024) and PRISM for macOS, version 10.4.2 (GraphPad Software, San Diego, CA, U.S.A.) were used for statistical analyses. Linear Mixed-Effects Models were fitted with the *lme4* package for R (Bates et al., 2003). The *buildmer* package (Voeten, 2019) automated preliminary stepwise selection of fixed and random effects. We used the *performance* package (Lüdecke et al., 2019) to obtain marginal and conditional R^2^ (Nakagawa and Schielzeth, 2013); Variance-inflation factors and random effects distribution were evaluated using the *check_model* function from the same package while simulation-based residual diagnostics were obtained with *DHARMa* (Hartig, 2016). Final models were fitted with Restricted Maximum Likelihood (REML) to obtain unbiased variance components, and estimated marginal means with contrasts were produced using *emmeans* (Lenth, 2017). *p*-values for EMMs and their pairwise contrasts were corrected for multiplicity using Hommel’s single-step procedure, with adjustments applied within each test family (e.g., all Publisher*PolCat3 comparisons constitute one family).

## 3 Results

For full results tables, see Supplementary Tables 1–3. The present study implemented repeated measures within observations: Initially, we measured subjective aesthetic appeal in a Restricted Exposure condition (*RatingRE*), a surrogate parameter for aesthetic evaluation under predominant activation of the perceptive processing channel. The follow-up measurement, taken in the Unrestricted Exposure condition (*RatingUE*), should therefore reflect the outcome after joint engagement of the perceptive channel and the slower cognitive processing channel. In the following sections, we first explore trends in our observed data, aggregated at the subject-level, asking the following questions:

(1) Do subject-level mean ratings differ between genders, political groups or stimulus categories when stimulus exposure is time restricted (*RatingRE*)?
(2) Do between-group differences emerge when exposure time is not restricted (*RatingUE*)?
(3) Do mean RE ratings significantly differ from mean UE ratings for specific groups (*RatingDELTA*)?
(4) Are the average within-observation changes (*RatingDELTA*) different between groups?

Second, we present the Core Model. Here, we address the following question:

(5) Out of the candidate variables considered, which ones are associated with between-exposure rating changes (*RatingDELTA*)?

Third, we analyze estimated marginal means (EMMs) derived from our Global Model. The questions addressed here are analogous to (3) and (4).

(6) Are between-exposure effects specific to certain participant-stimulus combinations?
(7) Does aesthetic reappraisal between-exposures (*RatingDELTA*) differ between specific groups?

For *post-hoc* comparisons between political groups, we chose to focus on LEFT–Right contrasts, as these represent distinct, opposite groups as defined by the political spectrum applied in the present study; The centrist group, conceptually an intermediate position rather than a distinct ideological pole and represented by markedly fewer participants than the Left- and Right-wing groups, was retained in the model to contribute to the pooled error term but was not considered for contrasts.

### 3.1 Observed subject-level mean responses

Initially, a comparison of the overall variances in observed subject-level mean (OSLM) ratings within each viewing condition revealed a significantly lower spread in the RE condition (*σ*^2^ = 585.80) than in the UE condition (*σ*^2^ = 824.37; *F* = 0.71, *p* < 0.001). This suggests that rapid, perceptual processing yields more uniform judgments, while prolonged engagement introduces additional variability, consistent with cognitive processing.

**Figure 7:**
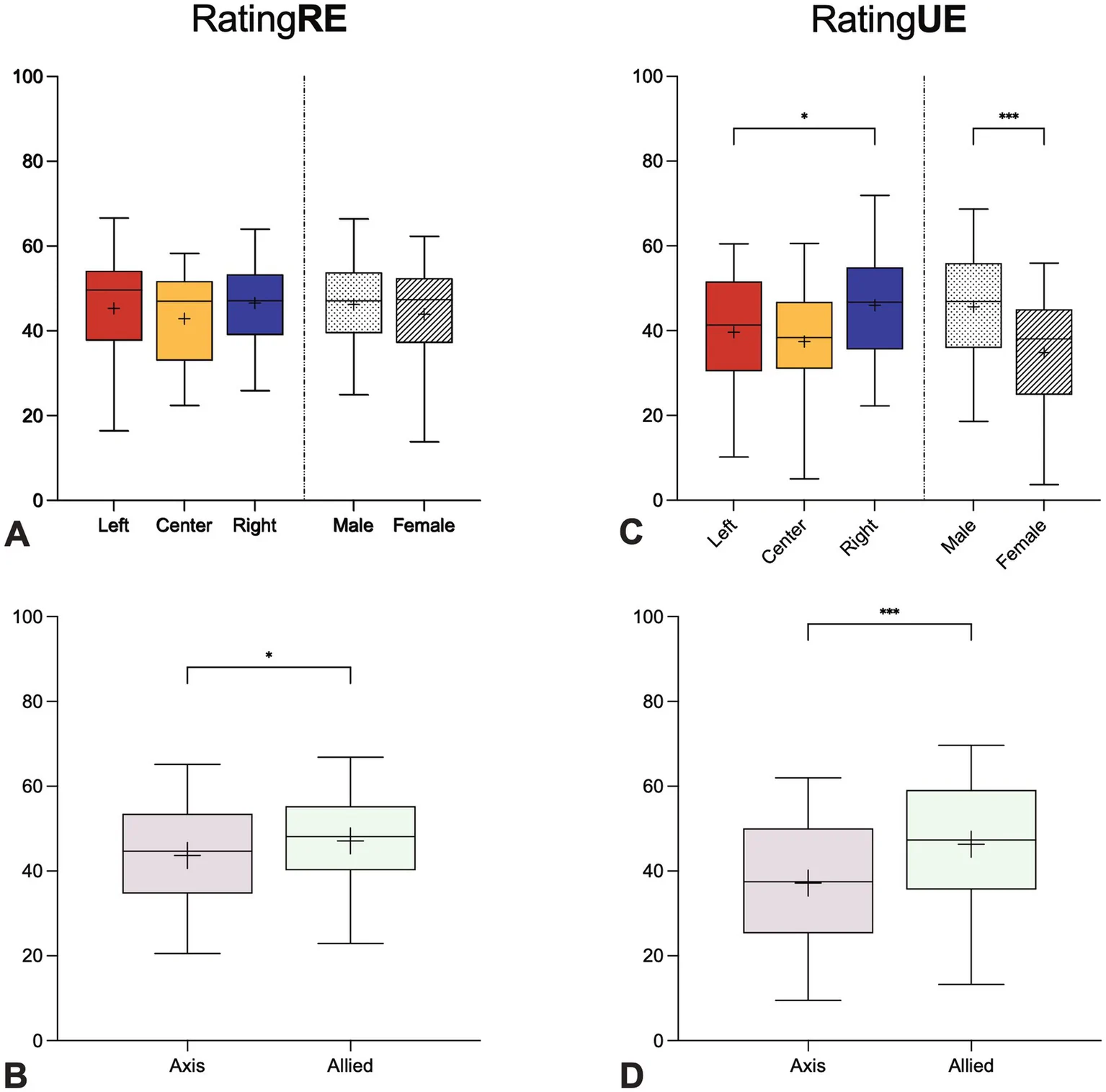
Comparisons of observed mean ratings in each exposure condition (rapid exposure = RE; unlimited exposure = UE): **(A,B)** show mean ratings per subject-group, compared between political groups and genders; **(C,D)** compare mean ratings between stimulus categories (publisher). Boxes represent the 25–75 percentiles, with mean (+) and median (horizontal line) shown. Whiskers indicate the 5–95 percentiles. Significance levels are *p < 0.05; **p < 0.01; ***p < 0.001.

Box plots in Figure 7 summarize subject-level mean ratings in each exposure condition by gender, political orientation (A and B) and, separately, by stimulus category (C and D).

First, we asked whether mean ratings differed between stimulus categories (Publisher) or participant-groups in the RE condition [question (1)]. At the stimulus level, an unpaired *t*-test comparing mean ratings by Publisher revealed that Axis stimuli were rated significantly lower than Allied stimuli (Mean Difference = −3.42 SE = 1.622, *p* = 0.036). Turning to subject-level comparisons, an additional unpaired *t*-test revealed no significant difference between male and female participants’ mean ratings (Mean Difference = −2.32 SE = 2.23, *p* = n.s.). Similarly, a one-way ANOVA detected no significant differences across the three political groups in our subject sample (*F* = 0.63, *p* = n.s.). These findings support our initial assumption that neither political alignment nor gender influence aesthetic appeal when exposure time and thus cognitive processing are restricted. The observed Publisher-difference, on the other hand, likely arose from differences in low-level image features rather than semantic context.

Next, we examined group-level differences in the UE condition [question (2)], applying the same analytical approach as in the RE condition. Here, female participants on average gave significantly lower ratings compared to males (Mean Difference = −10.79 SE = 2.56, *p* < 0.001). Regarding Political Orientation, we found significant differences between group means (*F* = 3,54, *p* = 0.032), with post-hoc tests showing that, on average, right-wing participants (Mean = 45.99 SE = 1.92) gave higher ratings compared to both Left-wing (Mean Difference = 6.33, *p* = 0.025) and centrist participants (Mean Difference = 8.75, *p* = 0.031). Finally, the difference in mean ratings between Publisher categories previously observed under RE became more pronounced in the UE condition, with Axis stimuli rated substantially lower than Allied ones (Mean Difference = −9.12, *p* < 0.001).

**Figure 8:**
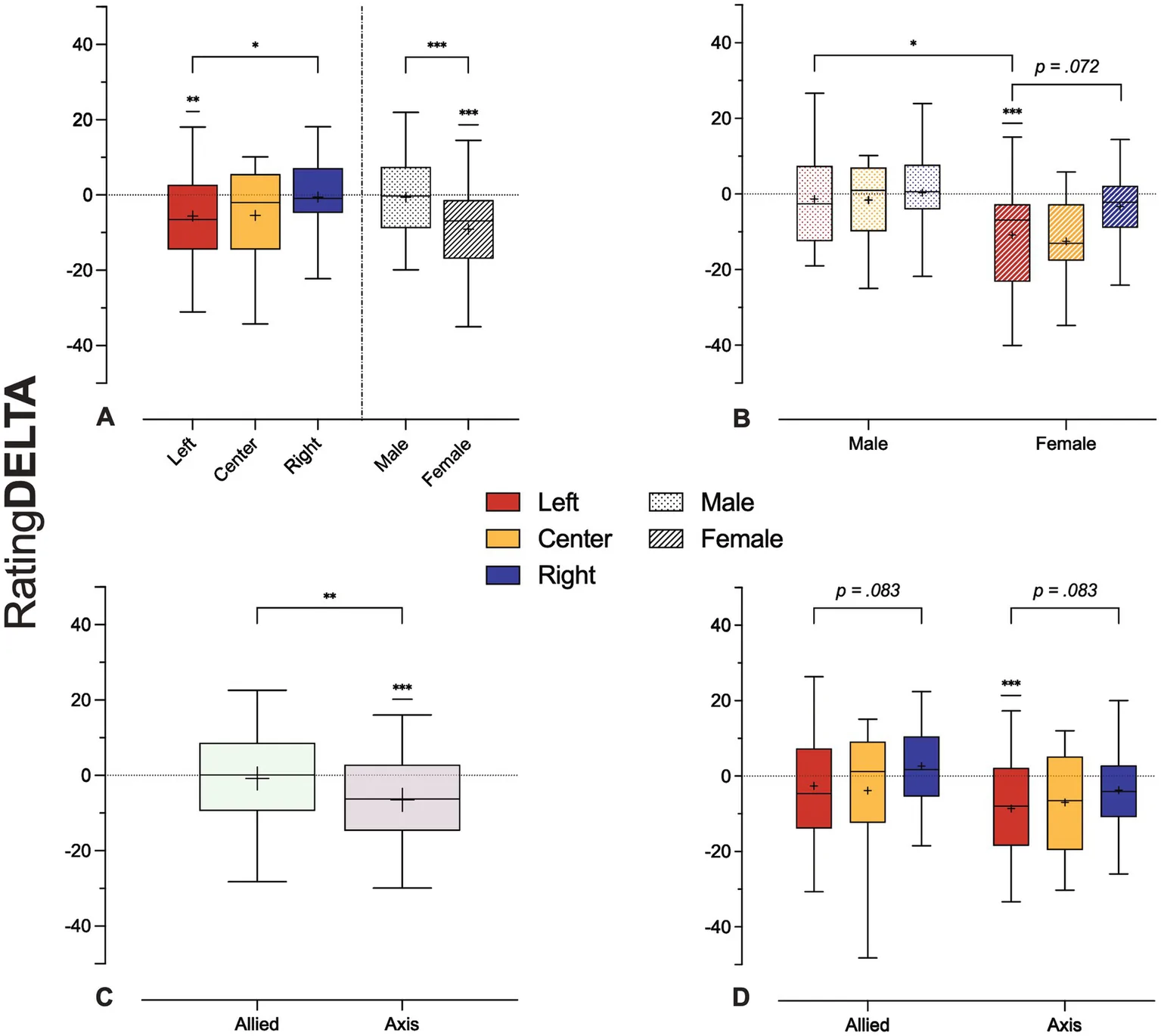
Comparisons of observed mean differences between exposure conditions (UE–RE): **(A)** comparisons between political groups and genders; **(B)** comparisons between political groups (*PolCat3*) within stimulus categories (*Publisher*); **(C)** comparisons between gender/political combinations; **(D)** Comparison between stimulus categories (*Publisher*). RatingDELTA = 0 indicates no change. Boxes represent the 25–75 percentiles, with mean (+) and median (horizontal line) shown. Whiskers indicate the 5–95 percentiles. Multiplicity-adjusted significance levels are ^*^p < 0.05; ^**^p < 0.01; ^***^p < 0.001.

For questions (3) and (4), we examined within-observation changes in ratings between exposure conditions [*RatingDELTA* (*Δ*) = *UE* − *RE*]. Figure 8 summarizes subject-level delta means by Political Orientation and gender (A), stimulus category (B), as well as Political Orientation stratified by Sex (C) and Publisher (D). First, we tested subject-group means across all stimuli against zero (Figure 8A). On average, Left-wing (*Δ* = −5.60, SE = 1.82, *p* = 0.003) and female participants (*Δ* = −9.06, SE = 1.92, *p* < 0.001) re-evaluated stimuli negatively.

A comparison of male and female group averages (Mean Difference = −8.47 SE = 2.22, p < 0.001) shows that female participants were more negative in their re-evaluation than male participants, with the overall average for males (*Δ* = −0.59 SE = 1.28, *p* = 0.643) indicating little change between exposure conditions.

Although an omnibus ANOVA across all three political groups did not reach significance (*F* = 2.40, *p* = 0.095), a planned contrast between Left- and Right-wing participants (Mean Difference = −5.06 SE = 2.42, *p* = 0.039) suggested that re-evaluation tended to be more negative in the left-wing group.

Given the considerable participant-overlap between the female and Left-wing groups, we stratified political groups by gender to isolate effects of political alignment from the overall gender effect (Figure 8C). Results hint at an interaction: Left-wing women showed a strong rating decrease (*Δ* = −10.81, *p* < 0.001), whereas no significant changes were observed in other female and, notably, none of the male political cohorts. As a follow-up, we performed an ANOVA (*F* = 3.78, *p* = 0.003): *Post-hoc* tests comparing between male and female participants within political groups revealed a significant gender difference within the Left-wing group (Mean Difference = −9.53, *p* = 0.003, *q* = 0.011). These findings suggest that the overall effects observed for the female and Left-wing cohorts are driven primarily by the Left-wing female sub-group. On the other hand, males did not differ significantly between political groups, and a comparison within the Right-wing cohort showed that males and females here, unlike in the Left-wing group, did not differ considerably.

The results by stimulus category (Figure 8D) aligned with earlier findings in the UE condition: On average, re-evaluation of Axis stimuli differed from re-evaluation of Allied stimuli and dropped significantly in subjective appeal (*Δ* = −6.51, SE = 1.22, *p* < 0.001), whereas ratings for Allied posters remained stable.

Next, we stratified by political groups (Figure 8B): For Axis posters, only Left-wing participants significantly changed their ratings between exposures (*Δ* = −8.59 SE = 1.96, *p* < 0.001). No significant changes were observed for Allied posters in any political group.

### 3.2 Model results

The following sections present the results of our Linear Mixed-Effects Models (LMMs). To provide a clear analytical picture, we distinguish between exploratory model development, followed by confirmatory and exploratory inferences derived from model-based EMMs.

First, we examine the Core Model (Section 3.3, Table 3 and Supplementary Tables 4, 5), an exploratory result of this study that provides the statistical framework for assessing the contribution of individual predictors to explained variance. Second, we turn to confirmatory (Section 3.4) and exploratory inferences (Section 3.5) derived from model-based estimated marginal means (EMMs). The analysis of the primary interaction between Political Orientation and Publisher—tested via Global Model EMMs—is treated as confirmatory. In contrast, analyses of the Political-Gender interaction and subsequent gender-stratified models are considered exploratory, aimed at probing the role of gender as a potential moderator.

**Table 3:** Core model summary.

Model fit^b^ fixed effects pseudo-*R^2^* = 0.26|total pseudo-*R*^2^ = 0.51								
Fixed effects	Variable	*df* ^c^	*t*	Estimate	SE	95% CI		Sig.
						LL	UL	
	(Intercept)	165.46	−3.02	−4.62	1.53	−7.62	−1.62	0.003
	pmcGist	137.49	−21.68	−15.08	0.70	−16.45	−13.72	<0.001
Sex^d^	Female–Male	128.71	−3.15	−8.11	2.58	−13.16	−3.07	0.002
Political group^e^	Left	128.53	−0.60	−0.95	1.57	−4.02	2.13	0.548
	Right	128.64	1.85	3.20	1.73	−0.19	6.58	0.067
Stimulus publisher^d^	Axis–Allied	74.89	−2.75	−6.86	2.50	−11.76	−1.96	0.008
Stimulus properties	IntentRecruitment^e^	72.76	2.48	4.20	1.69	0.88	7.53	0.015
	IntentProductivity^e^	72.37	−0.18	−0.31	1.77	−3.78	3.16	0.861
	IntentWarning^e^	67.41	−2.39	−4.01	1.68	−7.29	−0.72	0.020
QIPs	H-Channel	74.17	1.77	2.29	1.30	−0.25	4.84	0.081
	V-Channel	79.83	0.87	1.46	1.67	−1.82	4.74	0.385
	Complexity	69.87	−1.86	−1.96	1.05	−4.02	0.10	0.066
	Self-Similarity	72.92	1.49	1.60	1.07	−0.50	3.70	0.141
Interaction effects^e^	Axis*Left	926.07	−0.80	−0.52	0.65	−1.80	0.76	0.424
	Axis*Right	898.75	−2.19	−1.50	0.68	−2.83	−0.16	0.029
	Female*Left	128.24	−0.45	−0.70	1.57	−3.78	2.38	0.656
	Female*Right	127.44	1.26	2.17	1.72	−1.21	5.54	0.211
Linear mixed effects model fitted on RatingDELTA^a^								
^a^RatingDELTA = RatingUE − RatingRE. ^b^Model fitted on *n* = 2,700 observations by Restricted Maximum Likelihood (REML). ^c^Degrees of freedom calculated using the Satterthwaite method. ^d^Treatment contrasts: estimates represent difference between factor levels. ^e^Effect contrasts: estimates represent deviation from the grand-mean of the factor. ^f^Quantitative Image Properties.								

Random EffectsGroup	ICC	Slope	Variance	SD
Interview (*n* = 135)	0.21	Intercept	132.71	11.52			pmcGist	38.56	6.21			V-Channel	16.32	4.04	Stimulus (*n* = 80)	0.07	Intercept	43.96	6.63	Residual			457.53	21.39

Model estimates for sex and publisher are reported from the same model structure re-fitted with treatment contrasts for these variables. All other estimates use sum contrasts, interpreted at the grand mean of all variables.

As our study design presented random stimulus sets to participants, resulting in unequal exposition frequencies to the various stimulus-attributes, EMMs were computed from the Global Model, averaging over additional stimulus-level factors (i.e., Theme, Featured, Intent). Through the application of weighting schemes available in emmeans, we obtained estimates for the interaction terms investigated and subsequent post-hoc comparisons either based on the true sample distribution (cell) or a hypothetical equal distribution (equal) of stimulus attributes. Further documentation for the global model is provided in the Supplementary material (see Supplementary Tables 6–8).

Pseudo-R2 was identical for both the global and core models: Both models explained 51% of the total variance in *RatingDELTA*, with the fixed effects component explaining 27%. The intraclass correlation coefficient (ICC = 0.21) for participants (Interview) reveals a relevant within-participant clustering effect, whereas observations within the same stimuli were correlated to a lesser degree (ICC = 0.07). In sum [question (5)], these results indicate that our models explain a significant proportion of the observed variance through their random effects structure, confirming that ratings depended considerably on the individual participant (i.e., ratings from one participant tended to be similar) and to some extent on the stimulus itself (i.e., ratings for individual stimuli were somewhat similar, but varied more within a given stimulus than ratings coming from one participant).

### 3.3 Core model

First, we address baseline and stimulus-level controls: A strong negative effect was observed for the subject-mean centered RE ratings (*pmcGist*), indicating that high initial ratings constrained the range for potential positive re-evaluation. However, model results indicate the overall effect of *pmcGist* varies considerably at the subject-level.

Regarding QIPs that were selected for the model, trends for *H-Channel* and *Complexity* were approaching significance. *V-channel* and *Self-Similarity* were retained to stabilize model variance, as their removal from the model introduced heteroskedasticity. The random slope for *V-Channel* captured substantial subject-level variance, suggesting individual differences in preference for image brightness. Additionally, re-evaluation was influenced by the Intent category: *Recruitment* posters generally increased in appeal, whereas *Warning* posters showed a negative trend, likely reflecting their respective positive and negative visual messaging.

Second, we focus on predictors of theoretical interest: here, we observed a significant negative effect for Axis compared to Allied stimuli. Corroborating our aggregate-level analysis, a significant difference in re-appraisal emerged between genders: Across political groups and stimulus categories, female participants downgraded their ratings significantly compared to males upon re-exposure. Although the Sex*PolCat3 interaction term was not statistically significant, its inclusion in the model was justified by significantly improved residual behavior, suggesting it captures meaningful variance in the data structure that the model should account for. Finally, while Political Orientation did not show a significant main effect, the Publisher*PolCat3 interaction term was significant in the model, providing the basis for confirmatory EMM analysis in the following section.

### 3.4 Estimated marginal means for the political interaction terms in the global model

We computed EMMs for the PolCat3*Publisher interaction term under a cell weighting scheme, to preserve the observed sample distribution of variables that were averaged over, as these include Sex – a key variable averaged over in this analysis – interacts with PolCat3 and is unevenly distributed across political groups, equal weighting would have disproportionately inflated the influence of underrepresented Sex×PolCat3 combinations (e.g., right-wing females) on the resulting estimates.

**Figure 9:**
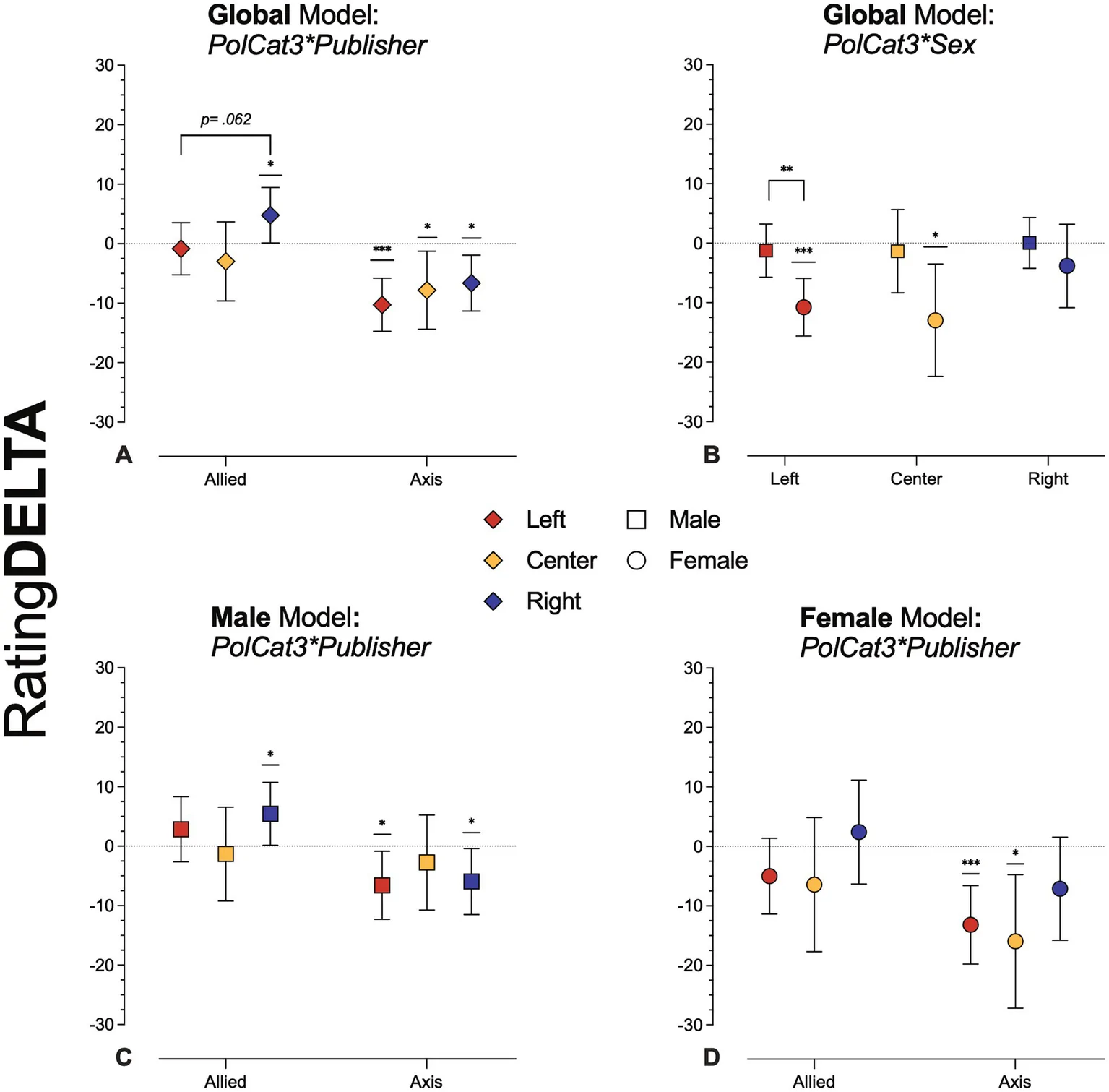
Model estimated marginal means for the interaction terms in the full model **(A,B)**, Male-stratified model **(C)** and Female-stratified model **(D)**. Symbols represent mean model estimates for deviation from 0 (= no change between Exposure conditions). The whiskers indicate the 95% CIs. Multiplicity-adjusted significance levels are *p < 0.05; **p < 0.01; ***p < 0.001.

Contrary to our previous observation on subject-level means, the model-estimated marginal means for the PolCat3*Publisher term (Figure 9A) show that participants in all three political groups rated Axis posters significantly lower in the UE condition. However, this trend was more robust in participants who self-identified as Left-wing (Estimate = −10.27 ± 2.26, *p* < 0.001), compared to the centrist (Estimate = −7.83 ± 3.33, *p* = 0.039) and right-wing (Estimate = −6.63 ± 2.38, *p* = 0.012) groups. By contrast, EMMs for Allied posters did not significantly differ from zero in all but the right-wing group, who, on average, re-evaluated these stimuli positively (Estimate = 4.78 ± 2.36, *p* = 0.045).

### 3.5 Exploring conditional gender effects

In a next step, we split the data into male and female subsets and re-fit the Global Model (omitting the Sex-term) on each subset separately (for model documentation, see Supplementary Tables 9–14). Even though we did not find sufficient empirical support for a three-way interaction between Sex, Political Orientation and Publisher during model selection, our final model did retain a two-way interaction between Sex and Political Orientation. For this term, EMMs were computed under an equal weighting scheme, treating levels of the stimulus-level factors averaged over (Publisher, Theme, Featured, Intent) as equally weighted to reflect a hypothetically balanced stimulus distribution. EMMs computed for the Sex*PolCat3 term (Figure 9B) closely resemble the pattern observed in our prior analysis at the aggregate level: Notably, results indicate a substantial negative between-exposure effect for Left-wing females (Estimate = 10.61 ± 2.65, *p* < 0.001), differing significantly from their Left-wing male counterparts (Estimate Difference = −9.51 ± 3.12, *p* = 0.009), for whom we did not observe an effect (Estimate = −1.25 ± 2.27, *p* = 0.583).

Considering this conditional gender effect, its strong main effect and the confounding risk posed by the considerable imbalance in the gender distribution across political groups (see Table 1), we concluded that, for accurate inference, the interaction between political context at the stimulus-level (*Publisher*) and subject-level political alignment (*PolCat3*) should be explored separately within male and female subsets. Thus, we ensure that estimates for the PolCat3*Publisher interaction term are not confounded by group-imbalances or masked by partial pooling, whereby underrepresented groups (e.g., right-wing females) have their estimates drawn toward the overall mean of all groups. However, as LMMs borrow strength for groups with few observations from the overall sample through the broader model structure (Gelman and Hill, 2006; Bryk and Raudenbush, 2002; Bates et al., 2015), stratification comes at the cost of reduced power.

Residual diagnostics indicated no signs of misspecification in either model (see Supplementary material). However, as the non-significant three-way Sex*PolCat3*Publisher interaction-term was eliminated from the Core/Global Model, findings presented in the following sections are strictly exploratory.

#### 3.5.1 Estimated marginal means for the Publisher*PolCat3 interaction in male participants

In the male-stratified model (Figure 9C), the Publisher*PolCat3 interaction remained statistically significant (*p* = 0.016), indicating systematic differences in rating changes for Axis versus Allied posters based on Political Orientation. Notably, right-wing men downgraded Axis posters significantly between exposures (Estimate = −5.95, *p* = 0.045), similar to their Left-wing counterparts (Estimate = −6.59, *p* = 0.049), with effect sizes and overlapping confidence intervals suggesting comparable rating behavior among these two groups. Centrist men, by contrast showed no reliable shift (Estimate = −2.58, *p* = 0.509), and their wide confidence interval reflects a high degree of uncertainty in responses from this group.

Notably, we observed a positive between-exposure effect for Allied in the right-wing group (Estimate = 5.43 ± 2.67, *p* = 0.044), suggesting that aesthetic appeal of Allied posters tended to improve specifically among right-wing males.

#### 3.5.2 Estimated marginal means for the Publisher*PolCat3 interaction in female participants

Contrary to what we observed in the male subset, the Publisher*PolCat3 was not significant in the female-stratified model—likely a result of the underpowered centrist and right-wing groups. Nevertheless, results (Figure 9D) show that both Left-wing (Estimate = −13.19 ± 3.35, *p* < 0.001) and centrist females (Estimate = −15.97 ± 5.78, *p* = 0.012) decreased aesthetic ratings for Axis posters between exposures, though there is considerable spread around the centrist estimate as indicated by the large standard error. By contrast, this effect appears to be robust in Left-wing females. Estimates for Allied posters suggest that ratings did not change significantly between exposures in any political group.

The results point to a consistent pattern of negative re-evaluation for Axis stimuli in the UE condition among Left- and centrist, but not right-wing females. However, the non-significant omnibus test of the interaction term, combined with considerable uncertainty around the centrist and right-wing female subgroups’ estimates mean that results should be interpreted with caution.

## 4 General discussion

This study examined how aesthetic judgements of political images—specifically, historical propaganda posters—change between rapid and unrestricted stimulus-presentation, depending on stimulus content, context and participant-level characteristics such as Political Orientation.

To interpret these effects, we draw from the UMAE (Redies, 2015) which proposes two parallel processing channels that determine aesthetic judgments: Rapid, perceptual processing of visual features (low-level), and slow, cognitive processing that integrates contextual information with domain-specific knowledge and a personal cultural filter. Our experimental design leveraged this framework by using repeated measurements under two exposure conditions: Restricted Exposure (RE), which primarily engages the perceptual processing channel, and Unrestricted Exposure (UE), which allows for prolonged engagement and recruits the slower cognitive processing channel. Here, we extend the UMAE into the political domain, proposing that Political Orientation is a part of the cultural filter during cognitive processing—shaping how observers engage with and reinterpret propaganda posters once their political message and context are recognized.

We predicted that the initial aesthetic judgements in the RE condition, driven by perceptual processing of visual features, would be relatively consistent across participants, whereas subsequent cognitive processing of semantic content and contextual information in the UE condition would significantly alter subjective aesthetic appeal based on participants’ political views. Consistent with our prediction, we found that overall spread in mean-ratings was significantly lower under RE compared to UE, indicating that the experiment successfully managed to isolate the rapid, perceptual processing channel in the RE condition. Conversely, increased variability observed in the UE condition, in our view, corresponds to activation of the more individualized cognitive processing channel modulating aesthetic experience.

Further, the strong negative relationship observed in our models between the covariate *pmcGist* (RE rating for a given observation adjusted for that participant’s overall rating tendency under RE) and the dependent variable *RatingDELTA* implies that rapid perceptual processing establishes a baseline judgment, from which aesthetic evaluations can then diverge.

Additionally, we predicted that RE ratings would not be affected by political views, whereas UE ratings would diverge depending on political alignment. Overall, results of the OSLM analyses aligned with the model-based EMMs, with both sets of analyses supporting an interaction effect between participants’ Political Orientation and stimulus-specific political context (PolCat3*Publisher).

Notably, a distinction emerged between OSLMs and the model-based EMMs regarding the re-evaluation of Axis posters: Contrary to OSLM analyses suggesting that only Left-wing participants re-evaluated these stimuli negatively, EMMs from the Global Model revealed that the centrist and right-wing groups similarly rated Axis posters significantly lower in the Unrestricted Exposure (UE) condition compared to Restricted Exposure (RE). Although this effect was more pronounced in Left-wing participants, Global Model EMMs suggest that re-evaluation of Axis posters was more universally negative across political groups, raising the question of which mechanisms underlie this effect. One possible explanation is that extended viewing enables access to semantic and ideological content, thereby engaging cognitively mediated evaluative processes (e.g., moral appraisal) that modulate initial perceptual judgments. Alternatively, the effect may reflect affective responses linked to historically negative associations, which become more salient with increased processing time. A further possibility is that the integration of perceptual and semantic information introduces processing disfluency, contributing to reduced aesthetic appeal. As these mechanisms were not independently manipulated in the present design, their relative contributions cannot be conclusively determined; however, the increased variability observed in the UE condition is consistent with the involvement of higher-order, individualized processing.

Mean ratings for Allied posters, by contrast, were generally consistent between exposures, with the exception of participants in the right-wing group, who actually improved upon their initial ratings.

These findings are consistent with the framework proposed under the UMAE. For instance, the uniformly negative re-evaluation of Axis posters illustrates the operation of a dominant cognitive channel that supersedes the perceptual channel, as collective memory and higher-order cognitive processing reinforce an overwhelmingly negative perception of Nazi Germany. It is therefore plausible that the most robust effect was observed in Left-wing participants, who are more likely to hold explicitly anti-fascist views. Likewise, increased appraisal of Allied posters in right-wing (specifically male) participants, could be explained by a preference for patriotic imagery. We hypothesize that resonance with the image’s semantic content fosters cognitive mastery through schema congruence, whereby alignment with the political context modulates aesthetic appeal.

Finally, we explored the effect of participant gender and its interaction with political orientation. Here, EMMs of the Sex*PolCat3 term in the Global Model revealed a marked difference specifically between male and female participants on the Left, with significantly more negative re-evaluations observed for females compared to male peers. Notably, this overall pattern of a gender-based divergence within the Left-wing, but not in other groups, directly mirrors the OSLMs.

Consequently, the detected Left-wing group estimate for the PolCat3*Publisher term in the Global Model may have been a result of the sizeable female cohort within this political group. To address potential confounding, we undertook an additional exploratory modelling-step by re-fitting the Global Model separately for male and female participants to obtain unbiased, gender-specific estimates of the two-way PolCat3*Publisher interaction. EMMs derived from these gender-stratified models indicated that, within each political group, male and female participants were trending in broadly similar directions, with some notable exceptions.

Within the male cohort, the right-wing group stood out for its positive re-evaluation of Allied posters, driving the overall right-wing group effect in the Global Model. Similar directional trends for Allied poster re-evaluation were observed in the female cohort—particularly among right-wing individuals—though these effects did not reach statistical significance. Notably, the female-stratified model showed a robust negative effect of Left-wing group membership on the reevaluation of Axis posters, whereas in the male-stratified model, both Left- and Right-wing groups downgraded Axis posters similarly.

Importantly, once the overall sex term was removed and gender was modelled separately, estimates for underrepresented subgroups were no longer biased towards the dominant political group within each gender (e.g., EMMs for the Right-wing and centrist groups in the female-stratified model were not drawn toward the mean of the large Left-wing subgroup). The nuanced political trends across genders support an effect of political orientation on cognitive processing, while also indicating that gender acts as a strong moderator in political-aesthetic evaluation. Interestingly, the observed gender-effect may be rooted in gender-specific biases toward aversive stimuli: In their meta-analysis of neuroimaging studies investigating whether brain activation to emotional stimuli differed between genders, Stevens and Hamann (2012) found that differences between men and women were highly dependent on the emotional valence of stimuli. Specifically, findings indicate that women exhibited greater activation for stimuli with negative valence, particularly in the amygdala, whereas men showed greater activation for positive emotion. In the context of the present study, the significant negative re-appraisal of Axis posters among female participants, particularly those in the Left-wing cohort, was conceivably driven by this heightened sensitivity to negative emotional valence, with Axis posters functioning as emotionally negative (i.e., aversive) stimuli – an impression possibly potentiated by their ideological opposition. By contrast, the positive re-appraisal of Allied posters by right-wing men aligns with a male-specific bias toward positive emotional stimuli. For this particular group, commonly featured themes of patriotism, military might and steadfast resolve likely functioned as pleasant emotional cues that resonated with their prior values, thus facilitating a gain in aesthetic appeal.

Beyond participant characteristics, stimulus properties made a modest contribution to aesthetic re-evaluation in our models. Recruitment posters generally increased in appeal, whereas Warning posters tended to decrease in appeal. This may reflect differences in semantic content: Recruitment posters often feature positive visual cues, whereas themes addressed in Warning posters (loose-lips, air raids) are reflected through mostly threatening imagery.

Fixed effects for Quantitative Image Properties (QIPs) were not significant in our models, but their inclusion improved overall model stability. This pattern aligns with the Unified Model of Aesthetic Evaluation (UMAE): After controlling for low-level image features, the change in aesthetic response appears to arise primarily from cognitive reappraisal rather than alterations in perceptual processing. In other words, longer viewing does not change how an image is perceived formally, but rather how it is understood.

## 5 Limitations

The present study highlights some of the challenges associated with research into the perceptive and cognitive mechanisms underpinning aesthetic experience. Limitations of this study are outlined below, alongside recommendations for future research.

First, several technical limitations arose from the online setting of our study, as this provided limited control over the experimental environment. Besides non-standardized screen size, resolution and colour display that may have inadvertently affected perception, true presentation time in the rapid exposure (RE) condition of the IRT was beyond our control and may have varied within the 500 ms window, a pragmatic compromise to ensure technical feasibility across different devices. However, we acknowledge that this duration may not fully isolate ‘pure’ gist perception, which is typically associated with exposure times closer to 100 ms. Consequently, some degree of higher-order processing may have been present during the Restricted Exposure condition, although the significant increase in overall rating-score variance between RE and UE demonstrates that isolation of cognitive and perceptual processing did occur.

Second, limitations related to participant sampling concern the gender distribution within political groups in our sample. Crucially, we did not anticipate the potential influence of gender and therefore did not control for group composition during sampling. This resulted in underrepresented and dominating subgroups (e.g., Left-wing dominance in the female cohort, underrepresentation of females in the right-wing group), producing large error margins and underpowered group comparisons. Although gender-stratified models partly address this limitation, the absence of significant interaction terms (Sex*PolCat3*Publisher, Sex*PolCat3 in the Global/Core model, and Publisher*PolCat3 in the Female-stratified model) should be interpreted with caution, as it may reflect limited statistical power to detect interaction effects in the presence of underrepresented groups. We recommend that future studies employ stratified sampling to balance gender distribution within political groups.

Another sampling-related limitation concerns the role of expertise: This factor (which we measured) was not considered for analysis due to its highly skewed distribution in our sample. However, prior research points to a significant role of expertise and semantic knowledge in aesthetic evaluation (Augustin and Leder, 2006; Else et al., 2015; Hayn-Leichsenring et al., 2022). It is therefore plausible that expert audiences, such as scholars in art or history, may engage differently with propaganda posters compared to laypersons. Future studies could address this by including participants from domain-specific expert populations (e.g., artists, historians, political scientists) in their sample.

Further consideration should be given to the definition of the political spectrum, which is subject to considerable cultural nuance, as participants from different countries may define political categories differently. Accordingly, defining personal political orientation on a Left–Right scale is constrained by the non-uniform meaning of ‘Left’ and ‘Right’ across contexts different national and cultural contexts. To address this limitation, future studies may benefit from collaboration with political scholars to develop dedicated questionnaires that provide a more objective measure of political orientation.

Our decision to work with authentic historical material posed additional challenges, as this approach inevitably restricted experimental control over image properties. One promising avenue for future research is the use of neural style transfer techniques (Geller et al., 2022) to generate custom-crafted stimuli. For example, poster designs associated with one political context could be emulated for another, allowing researchers to systematically manipulate artistic style, motif, and message to directly control image properties.

Finally, our modelling approach reflects the balance between complexity (to account for data hierarchy and interactions) with simplification (to ensure statistical feasibility). Along with previous recommendations for balanced sampling of subject and stimulus properties, ensuring a larger overall sample size will be crucial for improving power and enabling more reliable inference in the future.

## 6 Conclusion

Our study demonstrates that aesthetic evaluations of politically charged imagery, such as historical propaganda posters, are significantly influenced by both contextual exposure and individual viewer characteristics. Based on the Unified Model of Aesthetic Appreciation, we show that initial judgments driven by perceptual features in Restricted Exposure remain relatively stable across political groups, but Unrestricted Exposure presumably triggers cognitive processing that incorporates semantic content (i.e., political cues). We speculate that political alignment functions as part of the personal cultural filter, shaping how posters (in particular WWII propaganda posters) are re-evaluated. Notably, all political groups showed a decline in aesthetic ratings for Axis posters after Unrestricted Exposure, although this effect was most pronounced among Left-wing participants. Additionally, we observed a gender-based divergence in re-appraisal, with female participants on average downgrading ratings more than males, suggesting that gender moderates political-aesthetic evaluation. These findings highlight the dominant role of top-down cognitive processing in aesthetic evaluation of political content, revealing how political (dis-)congruence and gender interact with visual stimuli to shape subjective aesthetic experience.

## Data Availability

The raw data supporting the conclusions of this article are available at the website of the Open Science Framework (https://doi.org/10.17605/OSF.IO/GE576).

## Footnotes

1
https://www.prolific.com

2
https://www.soscisurvey.de
