# Supplementary material for: Partisan aesthetics: political orientation modulates aesthetic judgments of propaganda posters
Source: Front Psychol. 2026 Aug 12;17:1803013. doi: 10.3389/fpsyg.2026.1803013 (PMC13506364; doi:10.3389/fpsyg.2026.1803013)
Supplement: Supplementary file 1 [file Supplementary_file_1.pdf]

Supplementary Material

Model Selection

1 Data preparation

Linear mixed effects models (LMMs) were fitted on the original study data, thereby preserving information at the observation-level. Here, we directly model the between-exposure change (*RatingDELTA*) as a function of participant- and stimulus-level variables, thus providing a statistical framework that facilitates testing of our main hypothesis. Crucially, a LMM can be specified to accurately reflect the underlying data structure and hierarchy (see figure 5) through its random effects. Further, simulation studies have demonstrated that LMMs fitted on unbalanced data sets are generally robust, provided that data are missing completely at random (MCAR) with respect to the parameters and response of interest (Schielzeth et al., 2020). As our missing data in our experimental sample depend neither on covariate values (i.e. missing at random, MAR) nor the response variable or unobserved predictors (i.e. missing not at random, MNAR), the MCAR assumption holds in our data set. Following the recommendations in Yaremych et al. (2023) for hierarchical data structures, predictors were standardized prior to modeling: For *RatingRE,* a baseline covariate, we computed within-subject z-scores centered at the individual participant-means (i.e. the average of a participants' 20 RE ratings), thereby isolating within-subject deviations to control for different rating tendencies across participants. Stimulus-level predictors (Complexity, Anisotropy, Self-Similarity, H-Channel, S-Channel, V-Channel) were standardized at the grand mean of the stimulus pool.

2 Defining the Maximal Feasible Model from the Maximal Model

In a first step, we defined a Maximal Model (MM) that included all theoretically justified fixed- and random effects terms to control for all potential confounds (Barr et al., 2013; Harrison et al., 2018). We specified Interview (participant) and Stimulus as random-effects grouping factors to account for the crossed repeated-measures structure of the data, thereby modeling the within-group dependency of observations nested within participants and stimuli (Baayen et al., 2008). Predictors were then selected based on theoretical considerations and preliminary correlation analyses. However, the exploratory setting of our research limited our ability to make theoretically grounded assumptions that would have allowed further constraining the maximal model a priori. This in turn yielded a large set of theoretically

Supplementary Figure 1 | Flowchart of the Model Selection Process

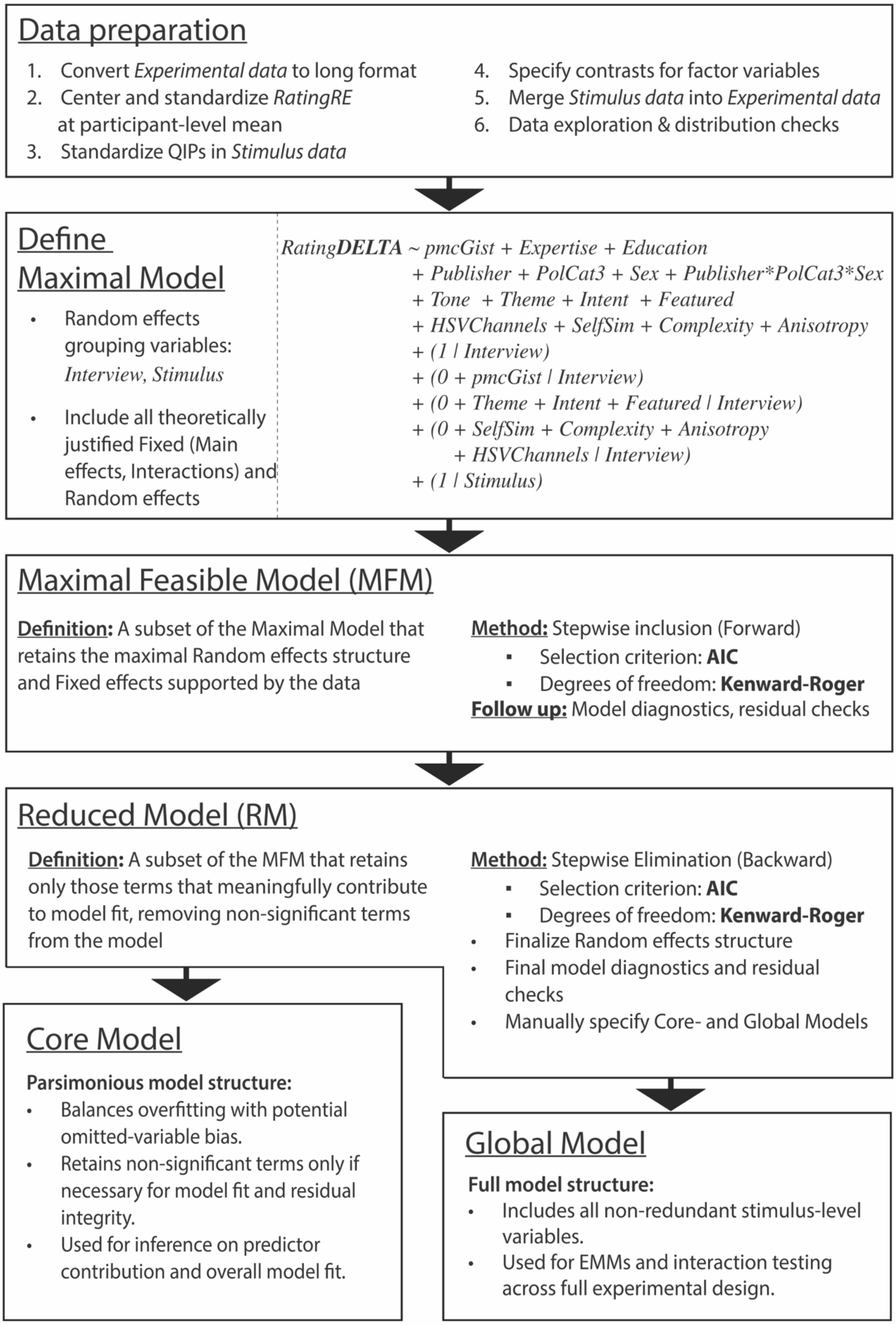

justifiable terms that were all included in the MM. The observational nature of our data further compounded the overparameterization issue, because factor levels of secondary variables such as *Background* (subject-level) or Stimulus properties, were not evenly distributed across our sample. As the number of parameters far exceeded what the data could reliably support, the maximal model ultimately failed to converge, a scenario where Bates et al. (Bates et al., 2018) and Matuschek et al. (Matuschek et al., 2017) argue that an appropriately parsimonious model be chosen instead.

To this end, we define the MM as a starting point from which we derive plausible subset models. For the fixed effects selection, we distinguished three groups of predictors: (i) Baseline control predictors, (ii) Exploratory stimulus-level predictors and (iii) Exploratory subject-level predictors. Fixed effects in group (i), including *pmcGist* and *Publisher*, were retained in every candidate model as baseline controls. Since any potential rating change between rapid (RE) and unlimited (UE) exposure is mathematically bounded by the starting (i.e. RE) score, we argue that candidate models should always control for this range-constraint. As for the *Publisher* variable, its central role in our experimental design and relationship with our outcome variables that emerged in analyses of subject-level means, necessitate inclusion of this main effect in all models. Variables in group (ii) encode information about stimuli that we regarded as potential modifiers, thus entering the entered the maximal model but were subject to elimination. Likewise, terms in group (iii) were allowed to compete freely in model selection as no prior evidence motivated specific constraints.

Rather than attempting to test all possible model subsets manually – an infeasible task given the number of plausible fixed, random and interaction terms – we automated the initial forward-selection using the *buildmer* function. This procedure identified the maximal feasible model (MFM): A beyond-optimal fixed effects structure (including interactions) upon which combinations of random slope terms were fitted, eventually arriving at the most comprehensive subset of the Maximal model that still converged. We used the Akaike Information Criterion (AIC) as the model selection criterion, in line with recommendations by Harrison et. al (2018).

3 Refinement and final model selection

Once the MFM was established, we derived the Reduced Model (RM) through a top-down strategy for refining the fixed effects-structure, as recommended by Zuur et al. (Zuur et al., 2009), using the Akaike Information Criterion (AIC) as the model selection criterion as we

did in the previous step. Non-significant fixed effects were iteratively removed to obtain a parsimonious model that preserved model fit and model-explained variance.

To arrive at the Core Model, we subjected the RM to final manual refinement of the fixed and random effects structures. At each step, we performed residual checks using DHARMa-simulated residuals to ensure that simplification did not come at the cost of assumption violations. In several instances, emerging heteroskedasticity or residual patterns observed after re-fitting a reduced model indicated that a removed term likely captured meaningful structure in the data, even if it was not statistically significant; In such cases, the predictor in question was retained, as its presence improved the overall model fit and stability (Hartig, 2024). For instance, we re-introduced an interaction term between Political orientation (*PolCat3*) and *Sex* after it had been eliminated during automated model selection: Even though the term itself was not significant, residual heteroskedasticity observed in the Reduced Model indicated that it may have been missing a predictor or interaction term. Since our previous analyses of subject-aggregated data hinted at a conditional effect between these two variables, we tested this interaction term manually, interpreting the much improved residual behaviour as confirmation of our missing-predictor assumption.

Conversely, the inclusion of certain fixed or random effects – such as the subject-level Background variable – led to violations of model assumptions, including heteroskedasticity and non-normal residuals, thus justifying their exclusion. Model refinement guided by residual diagnostics mitigate the risk of misinterpretation posed by violations of model assumptions (Bates et al., 2018). Throughout model reduction we adhered to the principle of marginality: whenever an interaction term or random slope remained in the model, all of its lower-order components were retained regardless of their individual significance.

In a final step, we reintroduced correlations among random slopes for more realistic modeling of within-group covariance structures, as previous iterations, starting with the MM, had been fitted with all random-slope correlations set to zero. Correlations whose inclusion produced singular fits were permanently dropped. We then inspected the variance components with *rePCA*; random slopes whose variance was < 0.01 or whose inclusion triggered convergence warnings were removed one at a time until the variance–covariance matrix was full-rank.

Supplementary Figure 2 | Core Model Formula

Core Model

$$
\begin{aligned}
\mathit{Rating}\boldsymbol{DELTA} \sim\ & 1 + \mathit{pmcGist} + \mathit{Sex} + \mathit{Publisher} + \mathit{PolCat3} \\
& + \mathit{Publisher}{*}\mathit{PolCat3} + \mathit{Sex}{*}\mathit{PolCat3} \\
& + \mathit{Intent} \\
& + \mathit{Complexity} + \mathit{SelfSim} \\
& + \mathit{HChannel} + \mathit{VChannel} \\
& + (1 + \mathit{pmcGist} + \mathit{VChannel} \mid \mathit{Interview}) \\
& + (1 \mid \mathit{Stimulus})
\end{aligned}
$$

This led to the retention of correlated random slopes for *V-Channel* and *pmcGist*, which improved model fit, while the random slope for *Self-Similarity* and *Anisotropy* were eliminated due to negligible variance contribution (singular fit when forced to correlate). The resulting Core Model thus represents the end-point of exploratory model selection, establishing the statistical framework for later hypothesis testing. This framework controls for subject- and stimulus-level differences through the random effects structure and the confounding influence of relevant fixed effects predictors.

Supplementary Figure 3 | Global Model Formula

Global Model

$$
\begin{aligned}
\mathit{Rating}\boldsymbol{DELTA} \sim\ & 1 + \mathit{pmcGist} + \mathit{Sex} + \mathit{Publisher} + \mathit{PolCat3} \\
& + \mathit{Publisher}{*}\mathit{PolCat3} + \mathit{Sex}{*}\mathit{PolCat3} \\
& + \mathit{Intent} + \mathit{Theme} + \mathit{Featured} \\
& + \mathit{Complexity} + \mathit{SelfSim} \\
& + \mathit{HChannel} + \mathit{VChannel} \\
& + (1 + \mathit{pmcGist} + \mathit{VChannel} \mid \mathit{Interview}) \\
& + (1 \mid \mathit{Stimulus})
\end{aligned}
$$

These variables were added to ensure that estimated marginal means (EMMs) and post-hoc

contrasts were adjusted for the full complexity of the stimulus set, despite their non-robust fixed effects.

References

Baayen, R. H., Davidson, D. J., & Bates, D. M. (2008). Mixed-effects modeling with crossed random effects for subjects and items. *Journal of Memory and Language*, *59*(4), 390–412. https://doi.org/10.1016/j.jml.2007.12.005

Barr, D. J., Levy, R., Scheepers, C., & Tily, H. J. (2013). Random effects structure for confirmatory hypothesis testing: Keep it maximal. *Journal of Memory and Language*, *68*(3), 10.1016/j.jml.2012.11.001. https://doi.org/10.1016/j.jml.2012.11.001

Bates, D., Kliegl, R., Vasishth, S., & Baayen, H. (2018). *Parsimonious Mixed Models* (arXiv:1506.04967). arXiv. https://doi.org/10.48550/arXiv.1506.04967

Harrison, X. A., Donaldson, L., Correa-Cano, M. E., Evans, J., Fisher, D. N., Goodwin, C. E. D., Robinson, B. S., Hodgson, D. J., & Inger, R. (2018). A brief introduction to mixed effects modelling and multi-model inference in ecology. *PeerJ*, *6*, e4794. https://doi.org/10.7717/peerj.4794

Hartig, F. (2024, October 17). *DHARMa: Residual diagnostics for hierarchical (multi-level/mixed) regression models*. https://cran.r-project.org/web/packages/DHARMa/vignettes/DHARMa.html

Matuschek, H., Kliegl, R., Vasishth, S., Baayen, H., & Bates, D. (2017). Balancing Type I error and power in linear mixed models. *Journal of Memory and Language*, *94*, 305–315. https://doi.org/10.1016/j.jml.2017.01.001

Schielzeth, H., Dingemanse, N. J., Nakagawa, S., Westneat, D. F., Allegue, H., Teplitsky, C., Réale, D., Dochtermann, N. A., Garamszegi, L. Z., & Araya-Ajoy, Y. G. (2020). Robustness of linear mixed-effects models to violations of distributional assumptions.

Methods in Ecology and Evolution, *11*(9), 1141–1152. https://doi.org/10.1111/2041-210X.13434

Yaremych, H. E., Preacher, K. J., & Hedeker, D. (2023). Centering categorical predictors in multilevel models: Best practices and interpretation. *Psychological Methods*, *28*(3), 613–630. https://doi.org/10.1037/met0000434

Zuur, A. F., Ieno, E. N., Walker, N., Saveliev, A. A., & Smith, G. M. (2009). *Mixed effects models and extensions in ecology with R*. Springer. https://doi.org/10.1007/978-0-387-87458-6

Supplementary Tables

1 Observed subject-level means (OSLMs, paired t-tests)

Supplementary Table 1 | Results of paired t-tests for subject-level means of matched RE and UE ratings

Stimulus category	*df*	*t*	*M*	95% *CI*		Sig.[a]
				LL	*UL*	
Allied	134	-0.62	-0.82	-3.43	1.79	.535
Axis	134	-5.32	-6.51	-8.94	-4.09	< .001
Overall	134	-3.23	-3.67	-5.89	-1.45	.003

Note. M = mean difference between Unlimited Exposure (UE) rating and RE rating, negative values represent lower UE rating. *CI* = Confidence Interval; *LL* = lower limit; *UL* = upper limit
[a] *p-value adjustment: Hommel* method for three tests

Supplementary Table 2 | Results of paired t-tests for subject-level means of matched RE and UE ratings

Stimulus category	Sex	*df*	*t*	*M*	95% *CI*		Sig.[a]
					LL	*UL*	
Allied	*Male*	85	1.28	1.98	-1.10	5.07	.410
	Female	48	-2.55	-5.74	-10.27	-1.22	.056
Axis	*Male*	85	-2.22	-3.17	-6.02	-0.33	.088
	Female	48	-6.16	-12.38	-16.42	-8.34	< .001
Overall	*Male*	85	-0.46	-0.59	-3.13	1.94	.643
	Female	48	-4.73	-9.06	-12.91	-5.21	.001

Note. M = mean difference between Unlimited Exposure (UE) rating and RE rating, negative values represent lower UE rating. *CI* = Confidence Interval; *LL* = lower limit; *UL* = upper limit
[a] *p-value adjustment: Hommel* method for three tests

Supplementary Table 3 | Paired t-tests for subject-level means of matched RE and UE ratings

Stimulus category	Political group	*df*	*t*	*M*	95% *CI*		Sig.[a]
					LL	*UL*	
Allied	*Left*	63	-1.27	-2.62	-6.74	1.50	.471
	Center	19	-1.03	-3.85	-11.65	3.94	.628
	Right	50	1.49	2.63	-0.92	6.18	.430
Axis	*Left*	63	-4.39	-8.59	-12.49	-4.68	< .001
	Center	19	-2.23	-7.00	-13.56	-0.44	.189
	Right	50	-2.20	-3.72	-7.11	-0.33	.185
Overall	*Left*	63	-3.09	-5.60	-9.23	-1.97	.024
	Center	19	-1.99	-5.43	-11.15	0.29	.308
	Right	50	-0.36	-0.55	-3.59	2.49	.720

M = mean difference between Unlimited Exposure (UE) rating and RE rating, negative values represent lower UE rating. *CI* = Confidence Interval; *LL* = lower limit; *UL* = upper limit
[a] *p-value adjustment: Hommel* method for three tests

2 Model documentation

2.1 Core Model

Supplementary Table 4 | Core Model
Type III Wald *Chi-Square* Test of Fixed Effects

**Variable**	***χ2***	***df***	**Sig.**
(Intercept)	9.11	1	.003
pmcGist	469.88	1	<.001
Sex	9.92	1	.002
PolCat3	3.50	2	.174
Publisher	7.54	1	.006
Intent	8.90	3	.031
Complexity	3.12	1	.077
Self-Similarity	0.76	1	.382
H-Channel	3.48	1	.062
V-Channel	2.22	1	.136
*Publisher*PolCat3* [a]	6.11	2	.047
*Sex*PolCat3*	1.63	2	.442

Note. Significant tests indicate improved model fit after the predictor is added to the model
[a] Main effects for *Publisher* and *PolCat3* are not directly interpretable in the presence of a significant interaction term

Supplementary Table 5 | Core Model
Likelihood Ratio Tests of Random Effects

**Group**	**Random term**	**LRT**	***df***	**Sig.**
Interview	pmcGist	70.59	3	<.001
	V-Channel	20.90	3	<.001
Stimulus		110.08	1	<.001

2.2 Global Model

Supplementary Table 6 | Global Model
Type III Wald *Chi-Square* Test of Fixed Effects

Variable	*χ2*	*df*	Sig.
(Intercept)	6.81	1	.009
pmcGist	472.54	1	<.001
Sex	9.77	1	.002
PolCat3	3.52	2	.172
Publisher	8.83	1	.003
Theme	2.33	2	.312
Intent	10.01	3	.018
Featured	2.29	3	.514
H-Channel	2.84	1	.092
V-Channel	0.23	1	.635
Self-Similarity	3.59	1	.058
Complexity	3.72	1	.054
*Publisher*PolCat3* [a]	6.24	2	.044
*Sex*PolCat3*	1.6388	2	.441

Note. Significant tests indicate improved model fit after the predictor is added to the model
[a] Main effects for *Publisher* and *PolCat3* not directly interpretable in the presence of a significant interaction term

Supplementary Table 7 | Global Model
Likelihood Ratio Tests of Random Effects

Group	Random term	LRT	*df*	Sig.
Interview	pmcGist	64.44	3	<.001
	V-Channel	14.82	3	.002
Stimulus		93.33	1	<.001

Supplementary Table 8 | Global Model Summary: Linear Mixed Effects Model fitted on *RatingDELTA* [a]

**Model fit** [b]	Fixed Effects Pseudo-R^2 = .27 | Total Pseudo-R^2 = .51							
**Fixed Effects**	**Variable**	***df***	***t***	**Estimate**	***SE***	**95% *CI***		**Sig.**[c]
						LL	*UL*	
	(Intercept)	140.23	-2.61	-4.71	1.80	-8.25	-1.17	.010
	pmcGist	137.64	-21.74	-15.11	0.70	-16.48	-13.75	<.001
Sex [d]	*Female – Male*	128.54	-3.13	-8.07	2.58	-13.13	-3.01	.002
Political group [e]	*Left*	128.38	-0.58	-0.92	1.57	-4.00	2.17	.561
	Right	128.47	1.86	3.22	1.73	-0.17	6.61	.065
Stimulus Publisher [d]	*Axis – Allied*	67.20	-2.97	-7.63	2.57	-12.67	-2.60	.004
Stimulus Classifiers [e]	*ThemeMilitary*	60.59	-1.37	-2.11	1.54	-5.14	0.91	.176
	ThemeWorkforce	59.45	0.29	0.61	2.10	-3.51	4.72	.773
	IntentRecruitment	65.45	2.83	5.35	1.89	1.65	9.05	.006
	IntentProductivity	58.21	-0.25	-0.64	2.56	-5.67	4.38	.803
	IntentWarning	60.15	-2.17	-4.25	1.96	-8.09	-0.42	.034
	FeaturedSymbols	64.22	1.39	3.27	2.35	-1.34	7.89	.169
	FeaturedCombat	61.65	0.26	0.55	2.08	-3.54	4.63	.794
	FeaturedGadgets	55.38	-1.25	-3.96	3.17	-10.18	2.26	.217
QIPs [f]	*H-Channel*	69.27	1.69	2.28	1.35	-0.37	4.92	.096
	V-Channel	70.43	0.47	0.82	1.73	-2.56	4.20	.636
	Self-Similarity	64.15	1.89	2.19	1.16	-0.08	4.46	.063
	Complexity	63.15	-1.93	-2.09	1.08	-4.21	0.03	.058
Interaction Effects [e]	*Axis*Left*	922.07	-0.81	-0.53	0.65	-1.80	0.75	.420
	*Axis*Right*	895.75	-2.21	-1.51	0.68	-2.85	-0.17	.027
	*Female*Left*	128.06	-0.46	-0.72	1.57	-3.80	2.37	.649
	*Female*Right*	127.26	1.26	2.17	1.73	-1.21	5.56	.211

**Random Effects**	Group	*ICC*	Slope	Variance	*SD*
	Interview (n = 135)	0.21	*Intercept*	133.40	11.55
			pmcGist	38.32	6.19
			V-Channel	16.00	4.00
	Stimulus (n = 80)	0.07	*Intercept*	43.96	6.63
	Residual			457.53	21.39

Model estimates for *Sex* and *Publisher* are from a model fit with treatment contrasts for these variables. All other estimates represent deviation from the grand mean of respective factor (sum contrasts).

[a] *RatingDELTA = RatingUE – RatingRE*

[b] Model fitted on *n* = 2700 Observations by Restricted Maximum Likelihood (REML)

[c] *p*-values calculated using Satterthwaite degrees of freedom method

[d] Treatment contrasts: Estimates represent difference between factor levels

[e] Effect contrasts: Estimates represent deviation from the grand-mean of the factor

[f] Quantitative Image Properties

2.3 Sex-stratified Models

2.3.1 Male-stratified Model

Supplementary Table 9 | Male-stratified Model Summary: Linear Mixed Effects Model fitted on *RatingDELTA* [a]

**Model fit** [b]	Fixed Effects Pseudo-R^2 = .24 | Total Pseudo-R^2 = .48							
**Fixed Effects**	**Variable**	***df***	***t***	**Estimate**	***SE***	**95% *CI***		**Sig.** [c]
						LL	*UL*	
	(Intercept)	119.96	-0.65	-1.25	1.92	-5.01	2.51	.515
	pmcGist	87.11	-16.73	-14.79	0.88	-16.52	-13.05	<.001
Political group [e]	*Left*	82.02	-0.26	-0.48	1.86	-4.13	3.17	.796
	Right	81.34	0.62	1.13	1.83	-2.46	4.71	.540
Stimulus Publisher [d]	*Axis – Allied*	70.61	-2.76	-7.42	2.68	-12.67	-2.16	.007
Stimulus Classifiers [e]	*ThemeMilitary*	61.71	-0.39	-0.63	1.60	-3.77	2.51	.696
	ThemeWorkforce	60.87	-0.37	-0.81	2.18	-5.08	3.46	.711
	IntentRecruitment	68.35	2.06	4.05	1.97	0.19	7.90	.044
	IntentProductivity	59.82	0.46	1.22	2.66	-4.00	6.44	.648
	IntentWarning	62.89	-2.33	-4.77	2.05	-8.78	-0.76	.023
	FeaturedSymbols	65.52	1.08	2.64	2.45	-2.15	7.44	.284
	FeaturedCombat	62.53	-0.18	-0.39	2.16	-4.63	3.84	.856
	FeaturedGadgets	54.88	-0.95	-3.10	3.27	-9.51	3.30	.347
QIPs [f]	*H-Channel*	73.72	1.78	2.53	1.42	-0.25	5.30	.079
	V-Channel	73.59	0.33	0.60	1.81	-2.95	4.15	.741
	Self-Similarity	64.29	-1.04	-1.17	1.12	-3.38	1.03	.300
	Complexity	65.48	1.70	2.05	1.20	-0.31	4.40	.093
Interaction Effects [e]	*Axis*Left*	517.46	-1.21	-1.01	0.84	-2.66	0.63	.228
	*Axis*Right*	519.82	-2.40	-1.98	0.82	-3.59	-0.36	.017

**Random Effects**	Group	*ICC*	Slope	Variance	*SD*
	Interview (n = 86)	0.19	*Intercept*	121.88	11.04
			pmcGist	39.44	6.28
			V-Channel	15.13	3.89
	Stimulus (n = 80)	0.06	*Intercept*	40.32	6.35
	Residual			467.86	21.63

Model estimate for *Publisher* from a model fit with treatment contrasts for this variable. All other estimates represent deviation from the grand mean of respective factor (sum contrasts).

[a] *RatingDELTA = RatingUE – RatingRE*

[b] Model fitted on n = 1720 Observations by Restricted Maximum Likelihood (REML)

[c] p-values calculated using Satterthwaite degrees of freedom method

[d] Treatment contrasts: Estimates represent difference between factor levels

[e] Effect contrasts: Estimates represent deviation from the grand-mean of the factor

[f] Quantitative Image Properties

Supplementary Table 10 | Male-stratified Model
Type III Wald *Chi-Square* Test of Fixed Effects

**Variable**	***χ2***	***df***	**Sig.**
(Intercept)	0.43	1	.514
pmcGist	279.99	1	<.001
PolCat3	0.41	2	.816
Publisher	7.64	1	.006
Theme	0.74	2	.691
Intent	7.40	3	.060
Featured	1.62	3	.655
H-Channel	3.19	1	.075
V-Channel	0.11	1	.737
Self-Similarity	2.90	1	.089
Complexity	1.09	1	.296
*Publisher*PolCat3* [a]	8.32	2	.016

Note. Significant tests indicate improved model fit after the predictor is added to the model
[a] Main effects for *Publisher* and *PolCat3* are not directly interpretable in the presence of a significant interaction term

Supplementary Table 11 | Male-stratified Model
Likelihood Ratio Tests of Random Effects

**Group**	**Random term**	**LRT**	***df***	**Sig.**
Interview	pmcGist	42.42	3	<.001
	V-Channel	8.41	3	.038
Stimulus		44.25	1	<.001

2.3.2 Female-stratified Model

Supplementary Table 12 | Female-stratified Model Summary: Linear Mixed Effects Model fitted on *RatingDELTA* [a]

**Model fit** [b]	Fixed Effects Pseudo-R^2 = .28 | Total Pseudo-R^2 = .53							
**Fixed Effects**	**Variable**	***df***	***t***	**Estimate**	***SE***	**95% *CI***		**Sig.**[c]
						LL	*UL*	
	(Intercept)	75.79	-2.76	-7.17	2.60	-12.27	-2.07	.007
	pmcGist	48.24	-13.77	-15.62	1.13	-17.84	-13.39	<.001
Political group [e]	*Left*	61.78	-2.81	-9.10	3.24	-15.46	-2.75	.007
	Right	45.59	-0.60	-1.55	2.57	-6.59	3.50	.551
Stimulus Publisher [d]	*Axis – Allied*	45.65	1.73	5.20	3.00	-0.68	11.07	.09
Stimulus Classifiers [e]	*ThemeMilitary*	48.95	-2.78	-5.11	1.84	-8.70	-1.51	.008
	ThemeWorkforce	47.10	1.20	2.97	2.48	-1.89	7.84	.237
	IntentRecruitment	61.79	3.33	7.80	2.34	3.22	12.39	.001
	IntentProductivity	44.94	-1.43	-4.24	2.97	-10.06	1.58	.16
	IntentWarning	47.87	-1.66	-3.79	2.29	-8.27	0.69	.104
	FeaturedSymbols	57.17	1.51	4.36	2.88	-1.28	9.99	.135
	FeaturedCombat	54.71	1.13	2.83	2.51	-2.09	7.75	.265
	FeaturedGadgets	48.06	-1.36	-5.01	3.68	-12.22	2.19	.179
QIPs [f]	*H-Channel*	67.88	0.61	1.03	1.70	-2.30	4.36	.545
	V-Channel	62.99	-0.04	-0.08	2.17	-4.33	4.17	.971
	Self-Similarity	58.29	-2.70	-3.57	1.32	-6.17	-0.98	.009
	Complexity	62.39	1.84	2.64	1.43	-0.17	5.45	.071
Interaction Effects [e]	*Axis*Left*	374.08	0.42	0.45	1.08	-1.66	2.56	.678
	*Axis*Right*	353.52	-0.17	-0.22	1.27	-2.71	2.27	.863

**Random Effects**	Group	*ICC*	Slope	Variance	*SD*
	Interview (n = 49)	0.23	*Intercept*	145.93	12.08
			pmcGist	36.48	6.04
			V-Channel	18.58	4.31
	Stimulus (n = 80)	0.06	*Intercept*	38.56	6.21
	Residual			443.95	21.07

Model estimate for *Publisher* from a model fit with treatment contrasts for this variable. All other estimates represent deviation from the grand mean of respective factor (sum contrasts).
[a] *RatingDELTA = RatingUE – RatingRE*
[b] Model fitted on *n* = 2700 Observations by Restricted Maximum Likelihood (REML)
[c] *p*-values calculated using Satterthwaite degrees of freedom method
[d] Treatment contrasts: Estimates represent difference between factor levels
[e] Effect contrasts: Estimates represent deviation from the grand-mean of the factor
[f] Quantitative Image Properties

Supplementary Table 13 | Female-stratified Model
Type III Wald *Chi-Square* Test of Fixed Effects

Variable	*χ2*	*df*	Sig.
(Intercept)	7.60	1	.006
pmcGist	189.52	1	<.001
PolCat3	3.08	2	.215
Publisher	7.88	1	.005
Theme	8.11	2	.017
Intent	12.89	3	.005
Featured	5.43	3	.143
H-Channel	0.37	1	.543
V-Channel	0.00	1	.971
Self-Similarity	3.39	1	.066
Complexity	7.27	1	.007
*Publisher*PolCat3* [a]	0.18	2	.914

Note. Significant tests indicate improved model fit after the predictor is added to the model
[a] Main effects for *Publisher* and *PolCat3* are not directly interpretable in the presence of a significant interaction term

Supplementary Table 14 | Female-stratified Model
Likelihood Ratio Tests of Random Effects

Group	Random term	LRT	*df*	Sig.
Interview	pmcGist	18.87	3	<.001
	V-Channel	4.97	3	.174
Stimulus		15.67	1	<.001

3 Model Estimated Marginal Means

Supplementary Table 15 | Global Model
Estimated Marginal Means for the *PolCat3*Publisher* Interaction Effect

Stimulus category	Political Group	*df*[a]	*t*-ratio	Estimate	*SE*	95% *CI*		*Sig.*[b]
						LL	*UL*	
Allied	*Left*	171	-0.38	-0.86	2.23	-5.25	3.54	.701
	Center	209	-0.89	-2.98	3.37	-9.62	3.66	.377
	Right	187	2.02	4.78	2.36	0.11	9.44	.045
Axis	*Left*	159	-4.54	-10.27	2.26	-14.73	-5.80	<.001
	Center	201	-2.35	-7.83	3.33	-14.39	-1.27	.039
	Right	182	-2.79	-6.63	2.38	-11.33	-1.94	.012

Note. Estimated marginal means for *RatingDELTA*, tested against 0 (= no change). Results are averaged over *Sex, Theme, Intent, Featured* using cell weights to represent true sampling distribution of participant- and stimulus-level variables.
[a] Degrees of freedom Method: *Satterthwaite*.
[b] Cross-group *p*-value adjustment*: Hommel* method.

Supplementary Table 16 | Global Model
Estimated Marginal Means for the *Sex* PolCat3* Interaction Effect

Stimulus category	Political Group	*df*[a]	*t*-ratio	Estimate	*SE*	95% *CI*		*Sig.*[b]
						LL	*UL*	
Male	*Left*	174	-0.45	-1.10	2.47	-5.97	3.77	.656
	Center	156	-0.48	-1.75	3.67	-9.01	5.51	.634
	Right	173	0.06	0.14	2.38	-4.55	4.84	.923
Female	*Left*	173	-4.01	-10.61	2.65	-15.83	-5.38	<.001
	Center	145	-2.61	-12.73	4.87	-22.36	-3.10	.020
	Right	157	-0.97	-3.58	3.69	-10.86	3.70	.666

Note. Estimated marginal means for *RatingDELTA*, tested against 0 (= no change). Results are averaged over *Publisher, Theme, Intent, Featured* using equal weights to represent a hypothetical equal sampling distribution across stimulus-level variables.
[a] Degrees of freedom Method: *Satterthwaite*.
[b] Cross-group *p*-value adjustment*: Hommel* method.

Supplementary Table 17 | Male-stratified Model
Estimated Marginal Means for the *PolCat3*Publisher* Interaction Effect

**Male-stratified Model Fit**[a] Fixed Effects Pseudo-R^2 = .24 | Total Pseudo-R^2 = .48 | Interaction p = .016 [b]								
**Stimulus category**	**Political Group**	***df***[c]	***t*-ratio**	**Estimate**	***SE***	**95% *CI***		***Sig.***[d]
						LL	*UL*	
Allied	*Left*	144	1.03	2.85	2.77	-2.62	8.33	.304
	Center	140	-0.33	-1.31	3.99	-9.19	6.57	.743
	Right	143	2.03	5.43	2.67	0.14	10.72	.044
Axis	*Left*	148	-2.27	-6.59	2.90	-12.32	-0.86	.049
	Center	142	-0.68	-2.74	4.04	-10.74	5.26	.743
	Right	145	-2.12	-5.95	2.80	-11.49	-0.40	.045

Note. Estimated marginal means for *RatingDELTA*, tested against 0 (= no change). Results are averaged over *Theme, Intent, Featured* using equal weights to represent a hypothetical equal sampling distribution across stimulus-level variables.
[a] Model fitted on n = 1720 Observations by Restricted Maximum Likelihood (REML)
[b] Type III Wald *chi-square* test
[c] Degrees of freedom Method: *Satterthwaite*.
[d] Cross-group *p*-value adjustment*: Hommel* method.

Supplementary Table 18 | Female-stratified Model
Estimated Marginal Means for the *PolCat3*Publisher* Interaction Effect

**Female-stratified Model Fit** [a]	Fixed Effects Pseudo-R^2 = .28 | Total Pseudo-R^2 = .53 | Interaction p = .916 [b]							
**Stimulus category**	**Political Group**	***df*** [c]	***t*-ratio**	**Estimate**	***SE***	**95% *CI***		***Sig.*** [d]
						LL	*UL*	
Allied	*Left*	94	-1.55	-4.98	3.25	-11.35	1.39	.124
	Center	74	-1.13	-6.41	5.81	-17.68	4.86	.261
	Right	84	0.55	2.43	4.49	-6.31	11.16	.582
Axis	*Left*	97	-3.98	-13.19	3.35	-19.77	-6.61	<.001
	Center	74	-2.84	-15.97	5.78	-27.19	-4.75	.012
	Right	86	-1.63	-7.12	4.46	-15.80	1.56	.214

Note. Estimated marginal means for *RatingDELTA*, tested against 0 (= no change). Results are averaged over *Theme, Intent, Featured* using equal weights to represent a hypothetical equal sampling distribution across stimulus-level variables.
[a] Model fitted on n = 980 Observations by Restricted Maximum Likelihood (REML)
[b] Type III Wald *chi-square* test
[c] Degrees of freedom Method: *Satterthwaite*.
[d] Cross-group p-value adjustment*: Hommel* method.

Model Diagnostics

1 Core Model

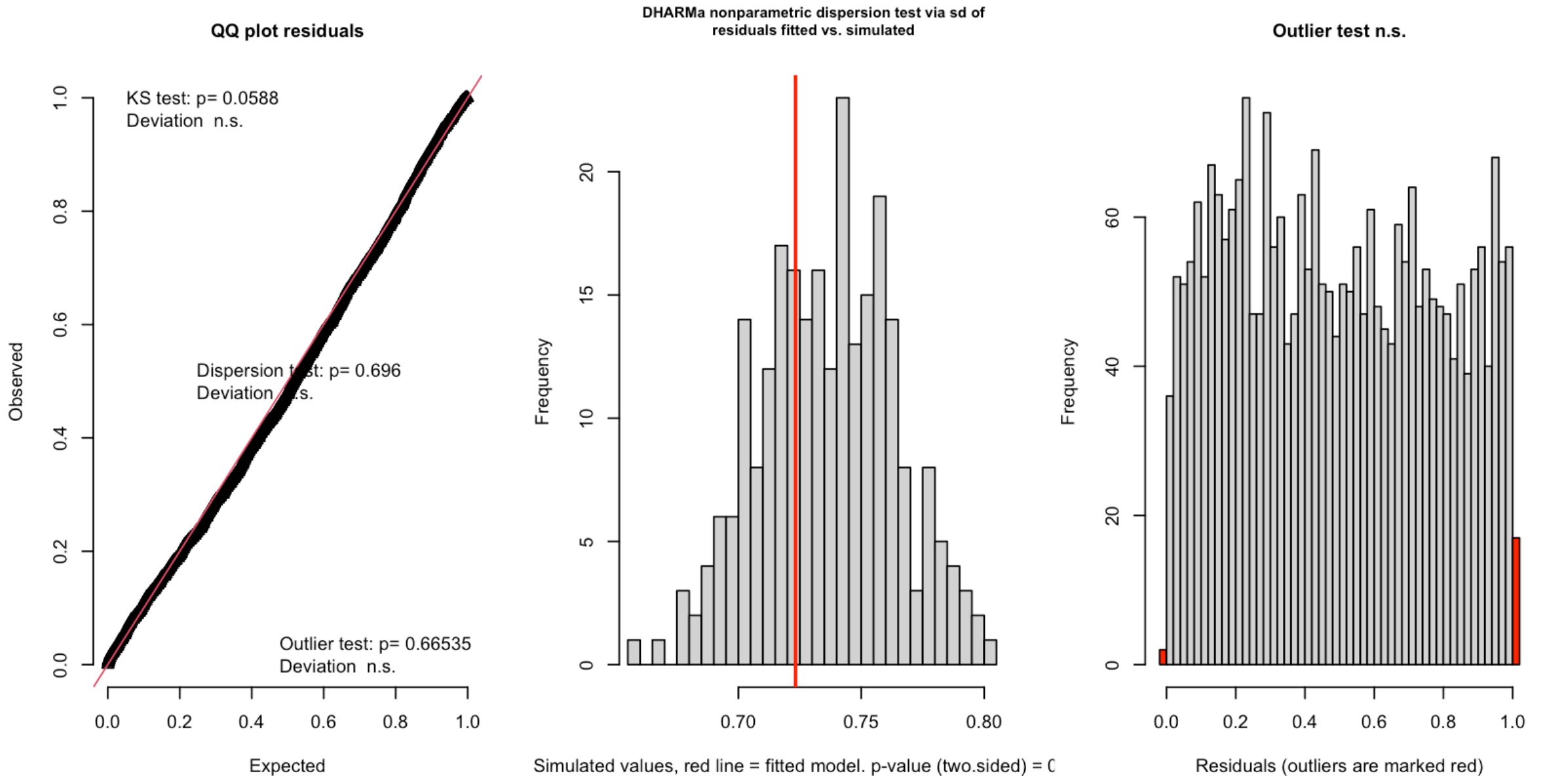

Supplementary Figure 4 | DHARMa diagnostic plots for the Core Model

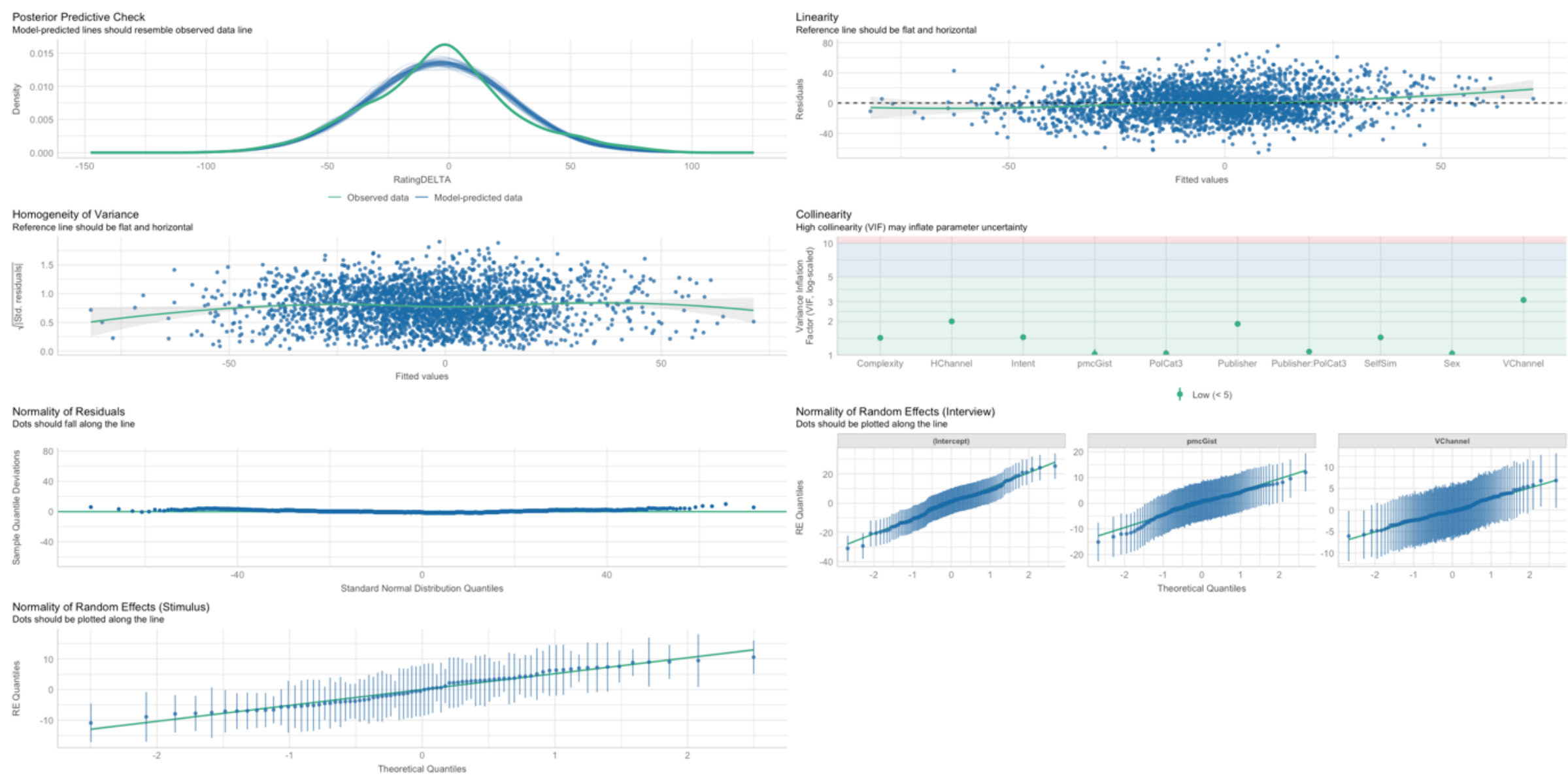

Supplementary Figure 5 | Diagnostic plots for the Core Model from the performance package

2 Global Model

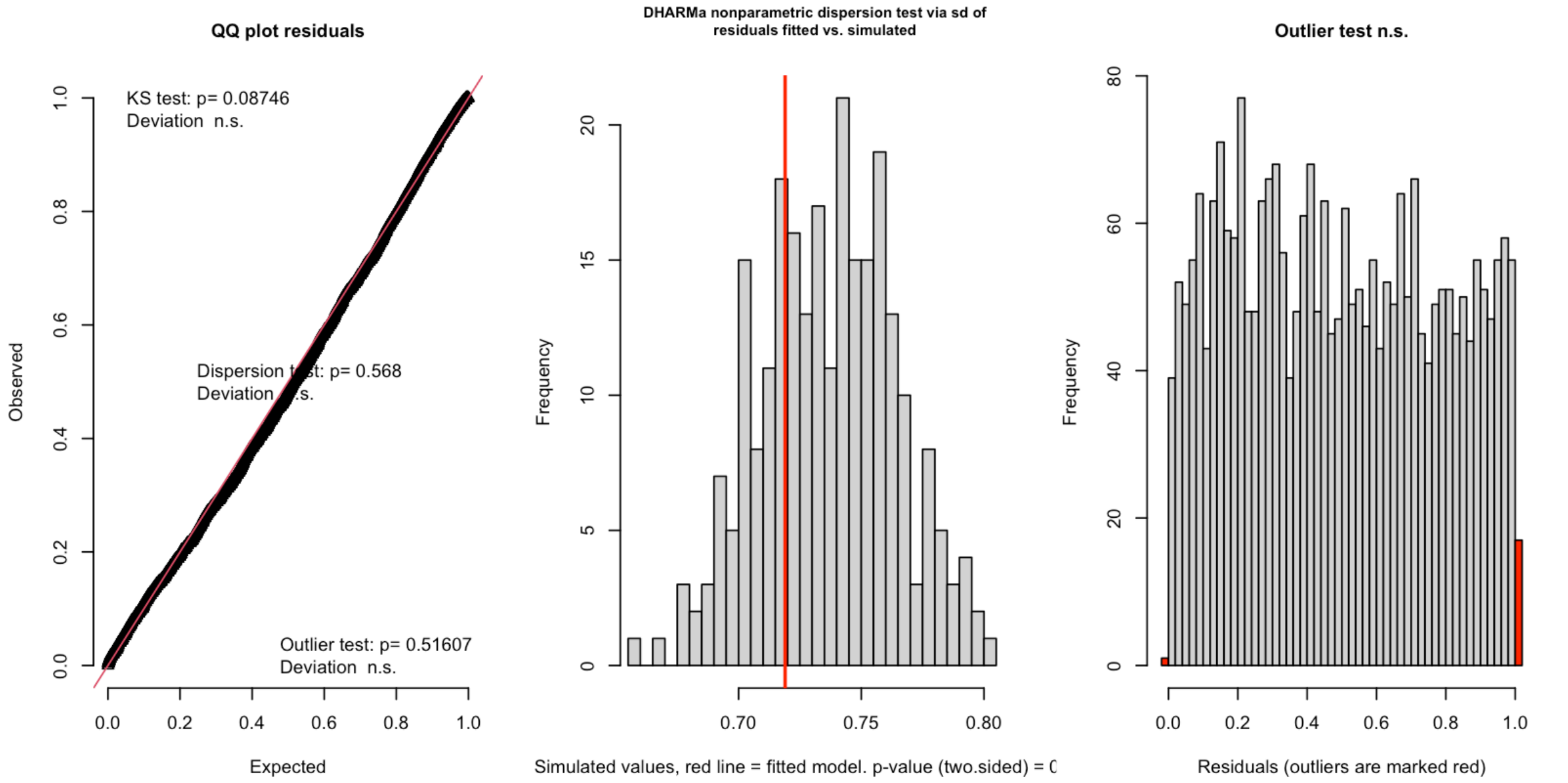

Supplementary Figure 6 | DHARMa diagnostic plots for the Global Model

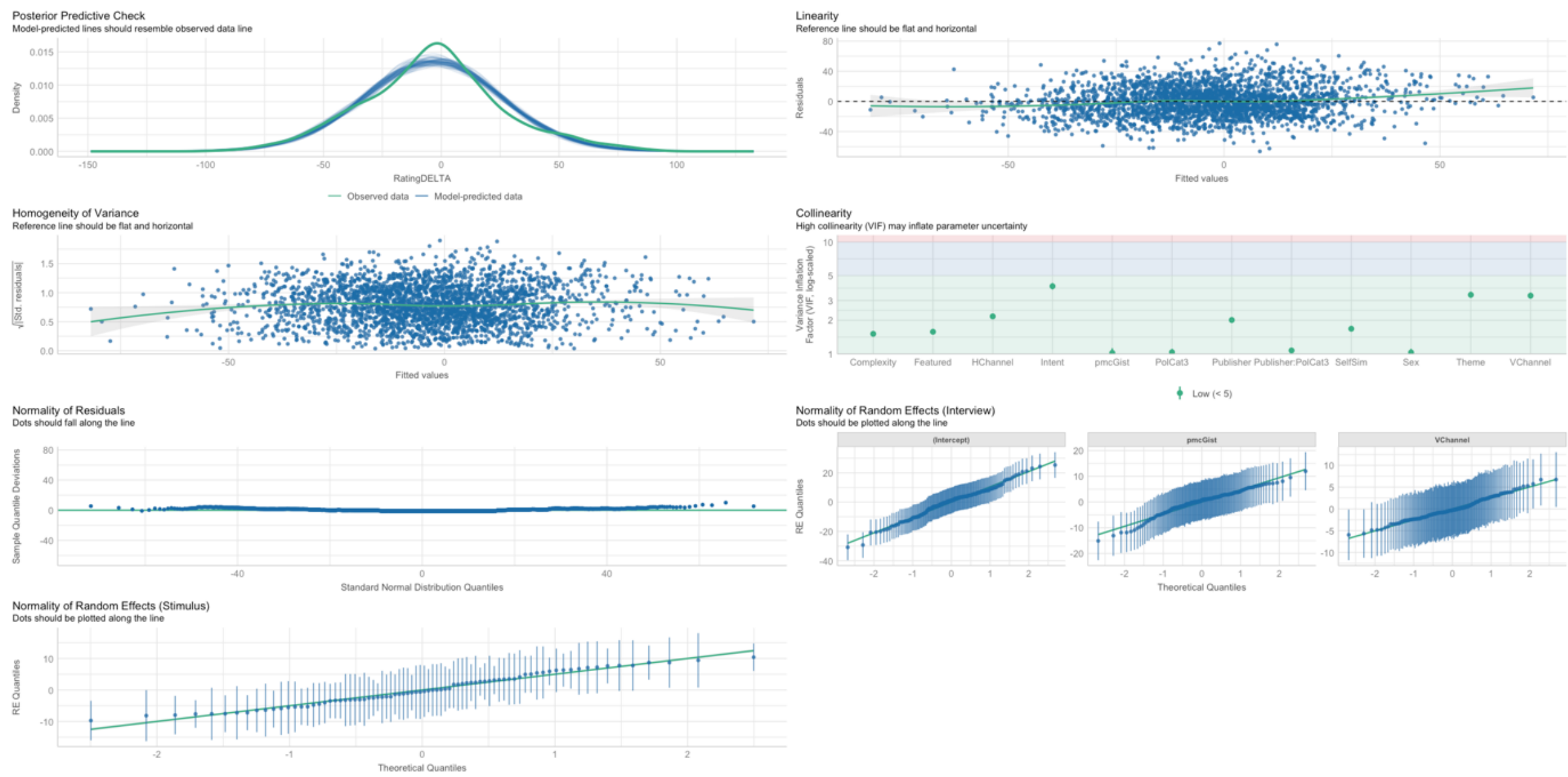

Supplementary Figure 7 | Diagnostic plots for the Global Model from the performance package

3 Male-stratified Model

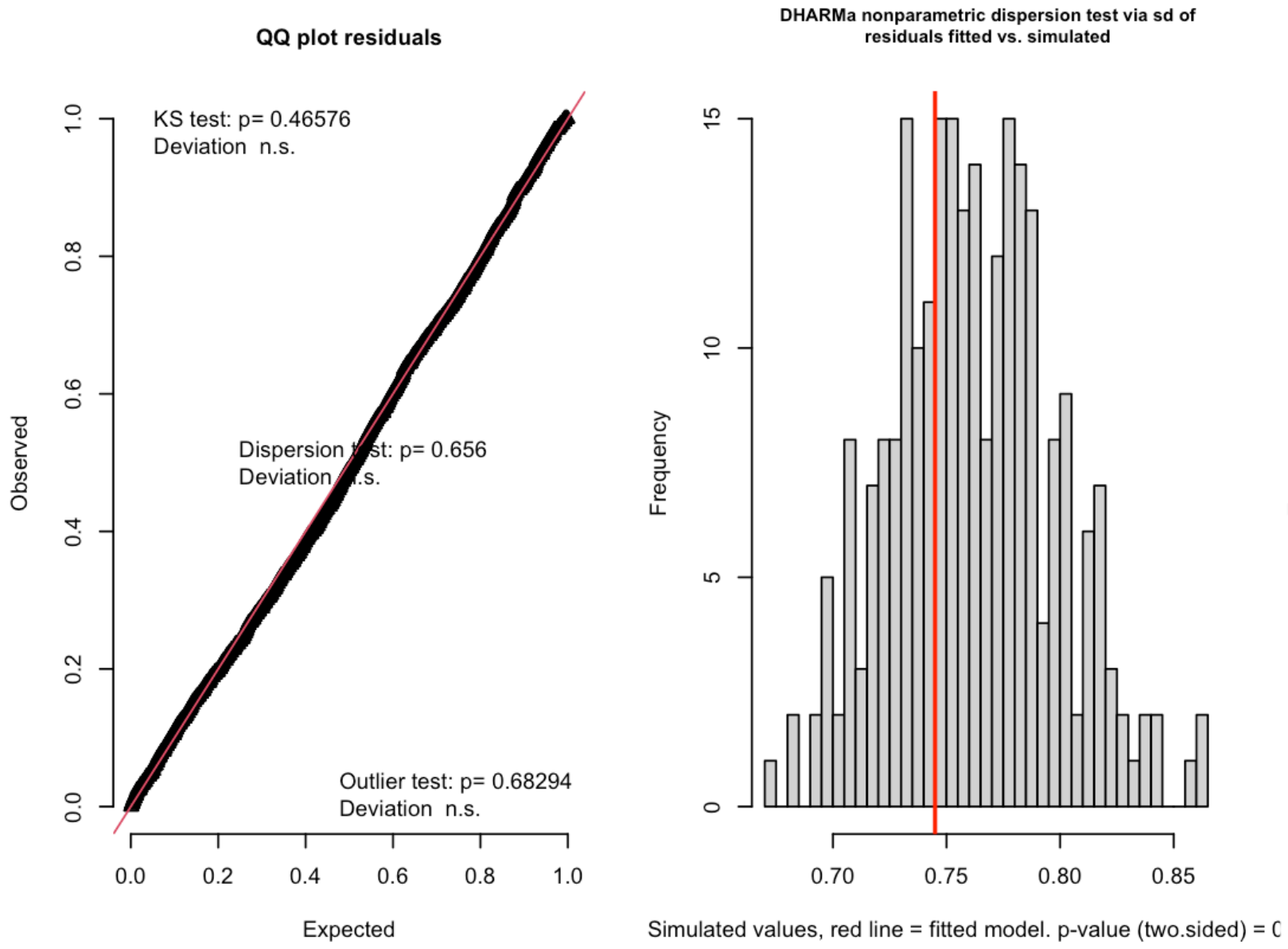

Supplementary Figure 8 | DHARMa diagnostic plots for the *Male*-stratified Model

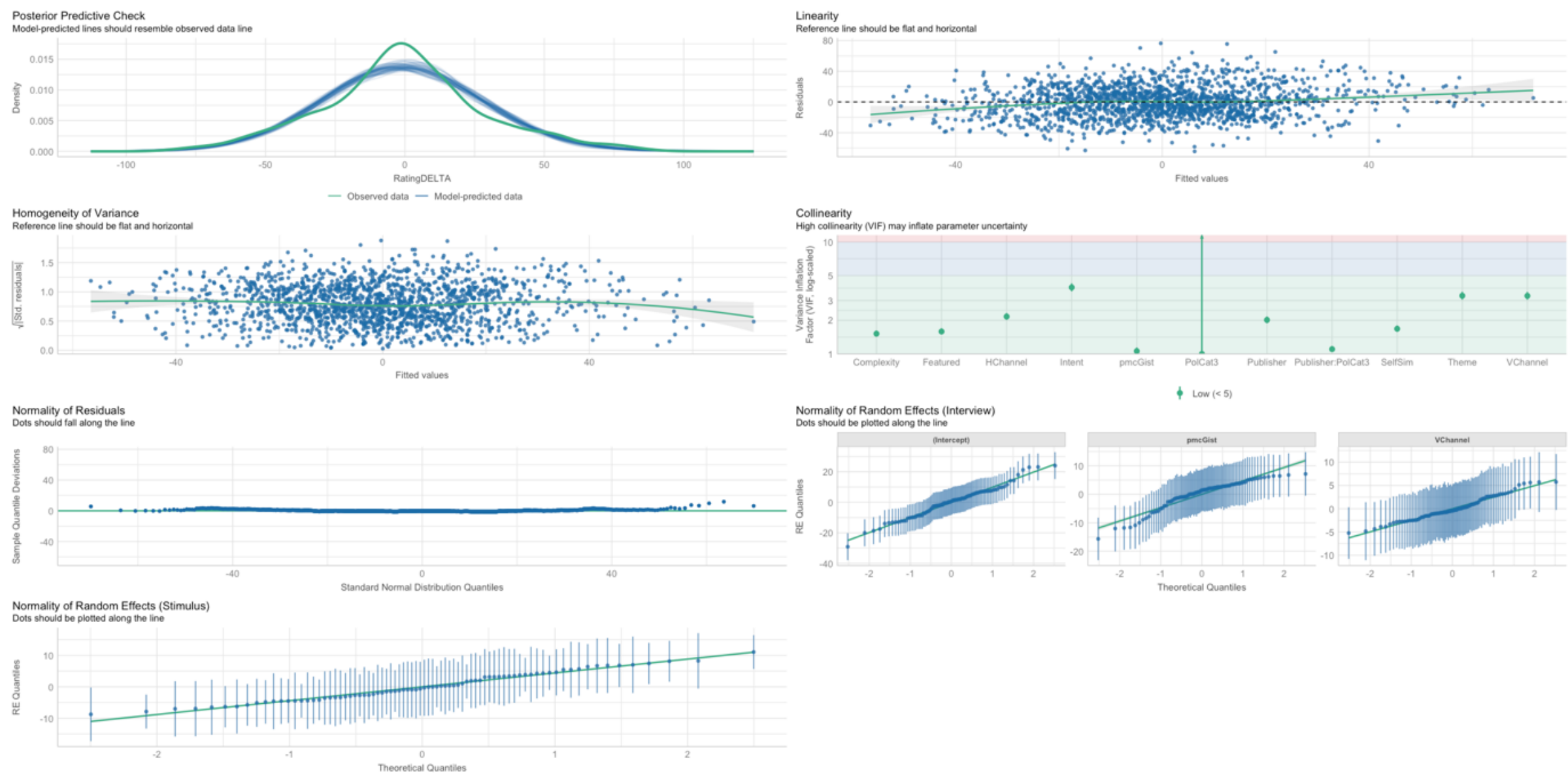

Supplementary Figure 9 | Diagnostic plots from the performance package for the *Male*-stratified Model

4 Female-stratified Model

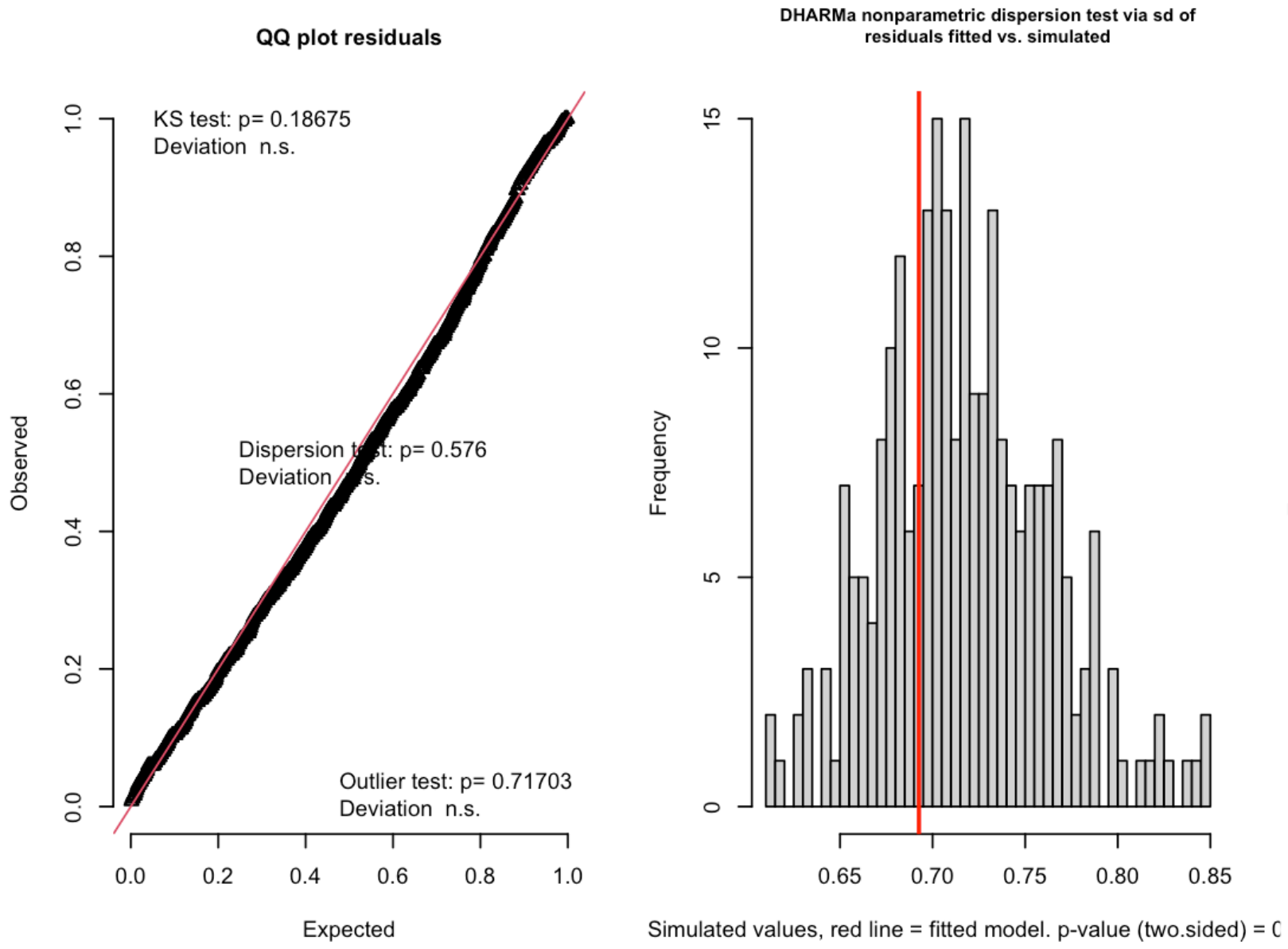

Supplementary Figure 10 | DHARMa diagnostic plots for the *female*-stratified Model

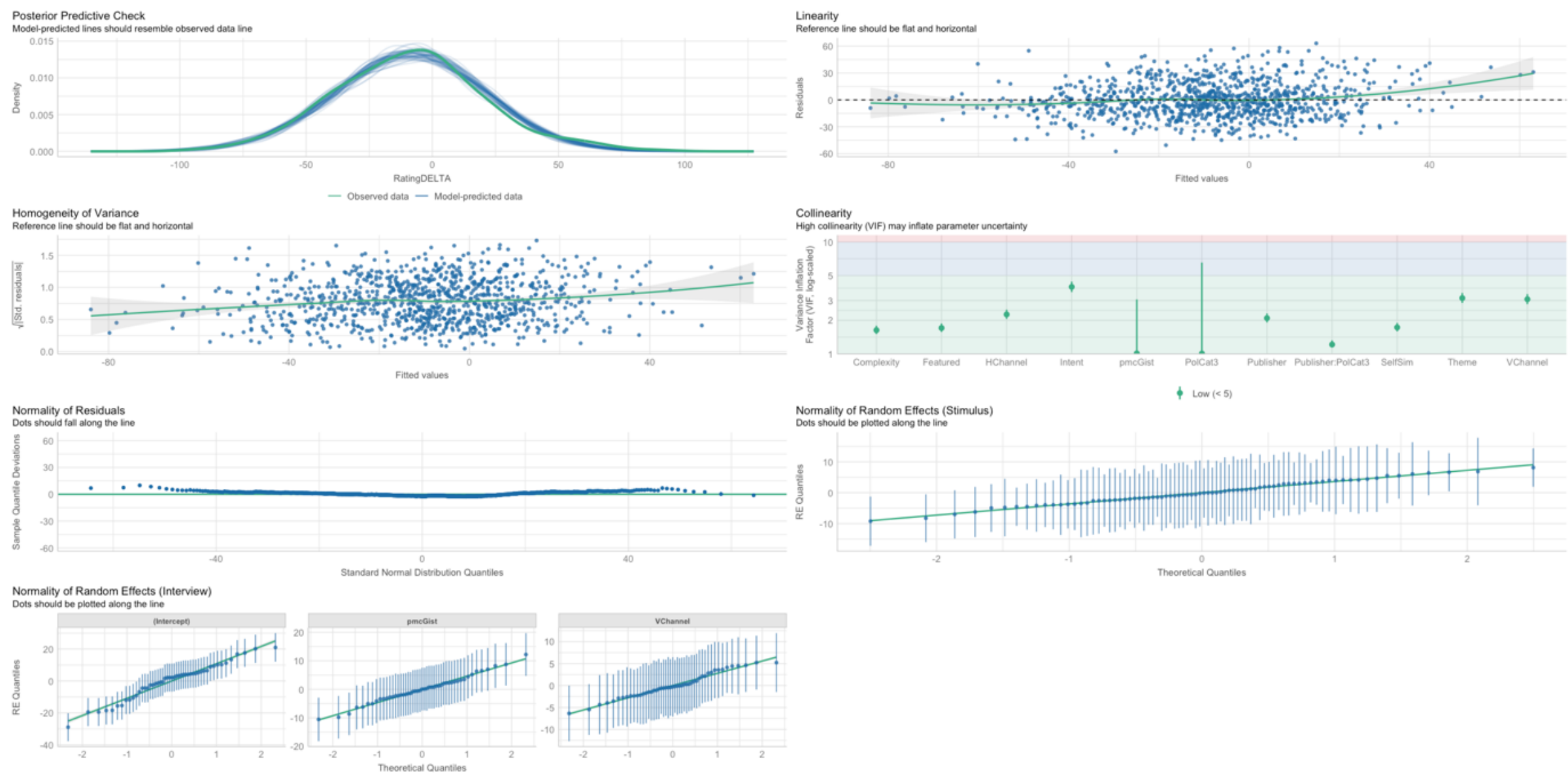

Supplementary Figure 11 | Diagnostic plots from the performance package for the *female*-stratified Model.
